# Cellular Energy Cycle Mediates an Advection‐Like Forward Cell Flow to Support Collective Invasion

**DOI:** 10.1002/advs.202400719

**Published:** 2024-06-21

**Authors:** Jian Zhang, Jenna A. Mosier, Yusheng Wu, Logan Waddle, Paul V. Taufalele, Wenjun Wang, Heng Sun, Cynthia A. Reinhart‐King

**Affiliations:** ^1^ Department of Biomedical Engineering Vanderbilt University 2301 Vanderbilt Place Nashville TN 37235 USA; ^2^ Department of Biomedical Engineering University of Arkansas 790 W. Dickson St Fayetteville AR 72701 USA

**Keywords:** bioenergetics, cancer metabolism, cell cycle, collective migration, go or grow, thermodynamics, tumor microenvironment

## Abstract

Collective cell migration is a model for nonequilibrium biological dynamics, which is important for morphogenesis, pattern formation, and cancer metastasis. The current understanding of cellular collective dynamics is based primarily on cells moving within a 2D epithelial monolayer. However, solid tumors often invade surrounding tissues in the form of a stream‐like 3D structure, and how biophysical cues are integrated at the cellular level to give rise to this collective streaming remains unclear. Here, it is shown that cell cycle‐mediated bioenergetics drive a forward advective flow of cells and energy to the front to support 3D collective invasion. The cell division cycle mediates a corresponding energy cycle such that cellular adenosine triphosphate (ATP) energy peaks just before division. A reaction–advection–diffusion (RAD) type model coupled with experimental measurements further indicates that most cells enter an active division cycle at rear positions during 3D streaming. Once the cells progress to a later stage toward division, the high intracellular energy allows them to preferentially stream toward the tip and become leader cells. This energy‐driven cellular flow may be a fundamental characteristic of 3D collective dynamics based on thermodynamic principles important for not only cancer invasion but also tissue morphogenesis.

## Graphical abstract

During 3D collective streaming, cells at the rear are more likely to enter an active cell cycle which also corresponds to a progressive increase in cellular adenosine triphosphate (ATP) energy. Once the cells progress to a later stage of the cell cycle toward cell division, the high intracellular energy allows them to preferentially stream toward the tip and become leader cells.

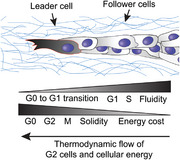

## 1 Introduction

Collective cell dynamics play crucial roles in morphogenesis and pattern formation during development, in tissue regeneration during wound response, in invasive diseases including excess angiogenesis and cancer, and in tissue engineering applications.^[^ ^1^, ^2^, ^3^, ^4^ ^]^ Many solid tumors often invade surrounding tissues in the form of multicellular 3D strands,^[^ ^5^, ^6^ ^]^ which exhibit distinct physical properties^[^ ^7^ ^]^ and collective dynamics^[^ ^8^, ^9^ ^]^ compared to the well‐studied 2D monolayer structure.^[^ ^10^ ^]^ Cell migration, like many other cellular processes, is long known to be driven by energy availability,^[^ ^11^ ^]^ especially when cells are navigating within a physically challenging 3D matrix.^[^ ^12^ ^]^ Minimization of thermodynamic cost is a governing principle that leader cancer cells use to invade 3D space.^[^ ^13^ ^]^ We recently demonstrated that during cancer collective invasion the leader cell consumes more energy than follower cells to do the work of paving the migration path forward, which depletes its available energy store. The energy‐depleted leader will be replaced by a more energetic new leader to sustain forward invasion.^[^ ^8^, ^12^ ^]^ This energy‐dependent “drafting” mechanism partially explains why tumor cell clusters are often more invasive and metastatic than individual tumor cells.^[^ ^14^ ^]^ However, it is not clear how high‐energy follower cells emerge to become the new leader.

Invasive migration and abnormal proliferation are two closely related hallmarks of cancer^[^ ^2^ ^]^ that together contribute to collective invasion and metastasis.^[^ ^15^, ^16^ ^]^ Their relationship has been studied for decades but remains unsettled. Some studies suggest a “go or grow” dilemma based on observations that cells in the frontal region are less proliferative than cells in the rear.^[^ ^17^, ^18^ ^]^ It was conjectured that due to the competition for shared resources, cells can only choose to either migrate or divide.^[^ ^19^ ^]^ However, many other studies support the opposite, showing highly migratory cells are also highly proliferative.^[^ ^19^, ^20^ ^]^ Additionally, a recent observation of single cells on 2D surfaces argued that migration and proliferation are independent of each other.^[^ ^21^ ^]^ These seemingly contradictory observations are largely due to a difference in cell types and the extracellular conditions in these studies, and a lack of mechanistic insight into the molecular players that connect cell migration and proliferation. Adenosine triphosphate (ATP) energy is one of the shared resources that connect migration and proliferation. Cell metabolism is tightly regulated during cell cycle progression for energy production and macromolecule synthesis via cyclin and cyclin‐dependent kinase (CDK) mediated signaling,^[^ ^22^ ^]^ such that cellular ATP production and availability may vary based on the cell cycle phase. However, it is not clear if and how this cell‐cycle‐regulated bioenergetics may feedback to affect cancer cell migration.

Here, based on iterative experimentation and modeling, we propose a biophysical model that connects cell cycle‐regulated bioenergetics to multicellular streaming. We found during stream‐like 3D collective migration, cancer cells in the rear of the invading strand have a higher probability than cells at the front to enter an active cell cycle phase (including G1 – gap phase for growth and preparation for DNA replication, S – DNA replication phase, G2 – gap phase preparing for mitosis, and M – mitosis phase for cell division) from the quiescent G0 phase, which decreases local density of G0 cells but increases that of G1 and S cells. Once entered an active cell cycle, cells progressively increase their ATP production, which was previously suggested as a means to prepare the cell for the progression through the cycle.^[^ ^22^ ^]^ We found this increased energy production is utilized by the migration machinery to meet the increased energy demand of forward migration, which drives an advective flow of high‐energy G2 cells toward the invasion front and supports leader invasion. Our findings demonstrate for the first time that the cell division cycle mediates a corresponding energy cycle, which feeds back to affect 3D cell migration, and G2 cells may be the most aggressive cells. These results provide the basis for understanding 3D collective dynamics based on thermodynamics‐related flow and for potential interventions to inhibit cancer collective invasion.

## 2 Results

### 2.1 Cellular ATP/Adenosine Diphosphate (ADP) Ratio Increases with Cell Cycle Progression

**Figure 1:**
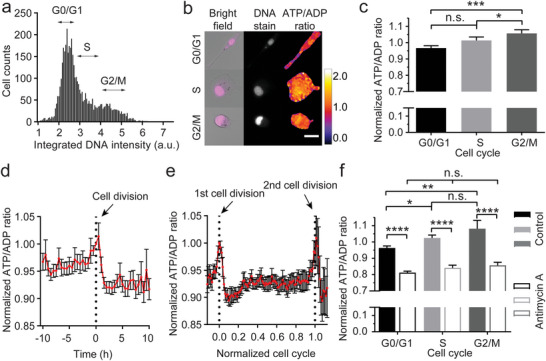
Cell cycle‐regulated bioenergetics. a) A histogram showing the typical distribution of cellular DNA amount in MDA‐MB‐231 cells labeled with the DRAQ5 far‐red DNA dye (from N = 4321 cells). The cell cycle phase (G0/G1, S, or G2/M) for each cell is determined from their integrated DNA stain intensity based on the histogram. b) Representative images of cells with simultaneous measurement of cell cycle phase from DNA stain and ATP/ADP ratio from the PercevalHR probe. c) Cellular energy level increases with cell cycle progression, with G2/M cells exhibiting the highest ATP/ADP ratio (N = 4394, 2168, and 1936 cells for each group, respectively; data normalized to the mean). d, e) Time‐lapse data shows that cellular energy level drops following cell division and then progressively increases until the next cell division (N = 92, and 18 cells, respectively; data normalized to the value at time/cell cycle = 0). Vertical dotted lines indicate the time of cell division. f) Inhibition of mitochondrial respiration by antimycin A eliminates the difference in ATP/ADP ratio between cells from different cell cycle phases (N = 2676, 3076, 1640, 1192, 697, and 1205 cells for each group, respectively; data normalized to the mean of control). Data are pooled from three independent experiments. Error bar represents S.E.M. Scale bar, 25 µm. Statistical significance is tested by the Kruskal–Wallis multiple comparison test with Dunn's correction. * *p* < 0.05, ** *p* < 0.01, *** *p* < 0.001, **** *p* < 0.0001, n.s. – not significant.

To elucidate the feedback from cell cycle progression to cellular energetics at a single cell level, we simultaneously measured intracellular ATP/ADP ratio using the PercevalHR probe and determined the cell cycle phase with the DRAQ5 far‐red DNA dye in MDA‐MB‐231 human breast cancer cells.^[^ ^8^ ^]^ By integrating the fluorescent intensity of the DNA dye over the nucleus, a histogram representing the distribution of cellular DNA amount was obtained (**Figure** **1**a). Gating values delimiting cell cycle phases were determined from the histogram and were then used to determine the cell cycle phase of each individual cell (Figure 1b), according to previous work.^[^ ^23^, ^24^ ^]^ Using this method, the cellular energy level was found to increase with cell cycle progression, with G2/M cells exhibiting the highest energy level in terms of ATP/ADP ratio (Figure 1c). Considering DNA‐content‐based cell cycle staging may be affected by genomic aberrations in cancer cells,^[^ ^24^ ^]^ we further confirmed the existence of such an energy cycle with time‐lapse confocal microscopy (Figure 1d,e; Figure S1a, Video S1, Supporting Information). This is consistent with the general consensus that proliferating cells produce and accumulate ATP energy during interphase (G1/S/G2 phase) in preparation for cell division.^[^ ^22^ ^]^ Mitochondrial respiration and ATP generation were previously reported to increase in the G2 phase due to phosphorylation of the mitochondrial chain complex I by the cyclin B1/CDK1 complex.^[^ ^25^ ^]^ Inhibiting mitochondrial respiration by antimycin A eliminated the energetic advantage of G2/M cells over cells in other phases (Figure 1f). By contrast, inhibiting glycolysis, another major pathway of cellular ATP production, by 2‐deoxy‐d‐glucose (2‐DG) was not able to diminish the energetic difference (Figure S1b, Supporting Information), although both treatments significantly decreased cellular energy level and proliferation when compared to control (Figure 1f; Figure S1b–d, Supporting Information). Together, our results suggest that cellular energy level is associated with cell cycle progression, with G2/M cells being the most energetic cells, which is likely caused by their increased mitochondrial respiration.

### 2.2 Differential Distribution of Cell Proliferation During Cancer Collective Invasion

**Figure 2:**
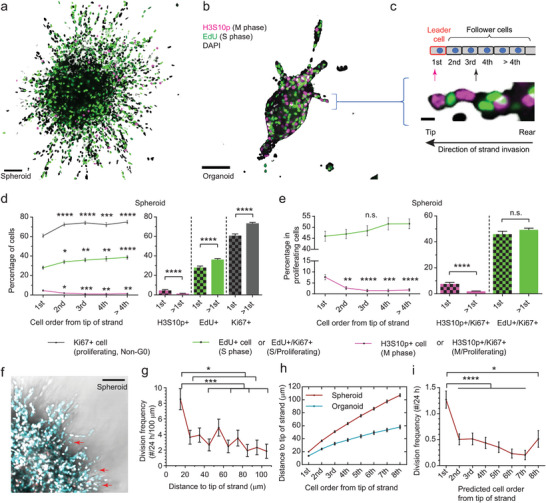
Differential regulation of cell cycle progression during cancer collective migration. a, b) Representative images of an MDA‐MB‐231 tumor spheroid and an MMTV‐PYMT tumor organoid invading in 4.5 mg mL^−1^ collagen with respective nuclear markers for proliferation. H3S10p, Histone H3 phosphorylated at Serine 10, a marker for M cell. EdU, 5‐ethynyl‐2′‐deoxyuridine, a nucleoside analog and a marker for S cell. c) An enlarged strand from (b) and a corresponding schematic showing the relative order of cells along a collectively invading strand, with the first cell being the leader cell. d) S phase (EdU+) cells and proliferating cells (Ki67+) are significantly enriched in follower positions, whereas M cells (H3S10p+) are more enriched in the leader position. Ki67 is a marker for all proliferative cells except those in the quiescent G0 phase. N = 796, 767, 632, 451, and 661 cells from 12 spheroids pooled from at least two independent experiments, respectively. e) The relative percentage of S cells compared to all proliferating cells is independent of the cell order along the strands, whereas the relative percentage of M cells is significantly enriched in the leader position. N = 485, 556, 469, 326, and 496 Ki67+ cells from 12 spheroids pooled from at least two independent experiments, respectively. f) A representative image of MDA‐MB‐231 spheroids expressing the CycleTrak cell cycle indicator (white indicates G1/G0 cells, cyan indicates S/G2/M cells) shows cell divisions (indicated by arrows). g) Normalized cell division frequency as a function of distance to the tip of the invading MDA‐MB‐231 strand. As the nuclear center rarely reaches within 10 µm distance to the tip, division frequency is not calculated in this range (data pooled from 20 spheroids from three independent experiments). h) Average distance to tip of invading strand as a function of cell order along the strand (N = 263 and 66 strands for spheroids and organoids, respectively, pooled from three independent experiments). i) Division frequency as a function of predicted cell order along invading MDA‐MB‐231 strands indicates the leader position has a higher division rate than follower positions. Statistical significance is tested by the Chi‐square test for independence, followed by multiple comparisons to the leader (first) group d, e), and comparison of Poisson means g, i). Significances for multiple pair‐wise comparisons are adjusted with the Bonferroni correction. Error bar represents S.E.M. Scale bar, 100 µm. * *p* < 0.05, ** *p* < 0.01, *** *p* < 0.001, **** *p* < 0.0001, n.s.—not significant.

We previously demonstrated that cellular energy level determines the migration state of leader cancer cells.^[^ ^8^ ^]^ To test whether the observed cell‐cycle mediated energy variation or the energy cycle also plays a role in collective invasion, we first investigated the distribution of cell cycle phases along collectively invading strands from tumor spheroids and organoids in 3D collagen^[^ ^8^ ^]^ (**Figure** **2**a,b). S, M, and non‐G0 proliferating cells were labeled with respective proliferation markers (Figure 2a,b, Figures S2a–d and S3a–d, Supporting Information), and their distributions were calculated for each location or order along the invading strands (Figure 2c). We found both proliferating cells and S cells were enriched at follower positions, or rear‐concentrated, in MDA‐MB‐231 spheroids in 4.5 mg mL^−1^ collagen (Figure 2d), consistent with previous reports that followers are more proliferative than leaders for endothelial cells^[^ ^26^ ^]^ and some cancer cells.^[^ ^18^ ^]^ Intuitively, more S cells should proportionally lead to more M cells, because once a cell enters an active cycle, it follows a clock to complete the cycle unless otherwise interrupted.^[^ ^27^ ^]^ Consistent with that, when quantifying the percentage of S and M cells relative to all proliferating cells, the relative distribution of S cells became flat (Figure 2e). Interestingly, however, M cells were highly enriched at the leader position, or front‐concentrated, instead (Figure 2d,e). Similar observations were confirmed with MDA‐MB‐231 spheroids in 3.0 and 6.0 mg mL^−1^ collagen matrices (Figure S2e,f, Supporting Information) and when cell cycle phases were determined from the Fucci4 probe instead of fluorescent staining^[^ ^28^ ^]^ (Figure S2g,h, Supporting Information), suggesting that the observation is not dependent on experimental conditions.

To test whether the above counterintuitive observation is caused by some specific molecular mechanisms in breast cancer cell lines, we first verified it using primary murine tumor organoids generated from MMTV‐PyMT mice.^[^ ^8^ ^]^ S phase and proliferating cells were more rear‐concentrated in organoids than in MDA‐MB‐231 spheroids with an approximately twofold enrichment in follower positions, whereas M cell distribution appeared flat in organoids (Figure S3e, Supporting Information). We speculated that the lack of M cell enrichment at the leader position in organoids may be attributed to the lack of proliferating cells there, so we quantified the percentage of S and M cells relative to all proliferating cells and confirmed the relative enrichment of M cells toward the leader position (Figure S3f, Supporting Information). Similar trends were also observed in spheroids composed of NIH3T3 mouse fibroblasts and 4T1 mouse breast cancer cells that also migrate collectively in the form of multicellular strands (Figure S3g–i, Supporting Information). By contrast, apparent differential trends for S and M phase cells were not observed at the wound edge of MDA‐MB‐231 and 4T1 monolayers on glass from a scratch wound healing assay (Figure S3j–l, Supporting Information) albeit both S and M phase cells appeared to be enriched toward the wound edge for 4T1 cells (Figure S3k, Supporting Information), suggesting that the differential distribution is specific for collective migration in the 3D strand form. Together, these data suggest that it is likely the physical microenvironment setup of 3D collective migration instead of a specific molecular program of breast cancer caused this differential distribution of cell proliferation.

To test whether the increased percentage of M cells at the leader position is caused by more cell divisions or elongated M phase duration there, cell division events along invading strands were tracked by time‐lapse confocal microscopy using MDA‐MB‐231 cells expressing the CycleTrak cell cycle indicator^[^ ^8^, ^29^ ^]^ (Figure 2f; Video S2, Supporting Information). Cell division frequency was found to decrease with increasing distance to the strand tip (Figure 2g; Figure S2i, Supporting Information). After converting the distance to cell order along the strand (Figure 2h; Figure S2d, Supporting Information), we confirmed a significantly higher cell division frequency at the leader position than at the follower positions (Figure 2i; Figure S2j, Supporting Information). This result clarified that the enrichment of M cells at the leader position is caused by increased division rather than delayed division.

### 2.3 Reaction–Advection–Diffusion (RAD) Model Predicts Forward Advection of G2 Cells

**Figure 3:**
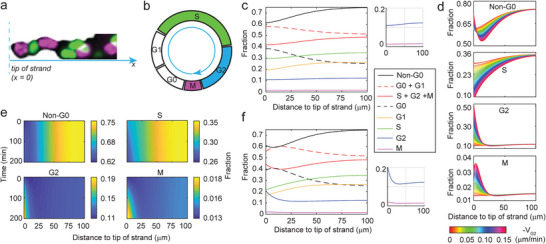
Computational modeling predicts the forward advective flow of G2 cells. a) Illustration of the 1D geometry used for the mathematical model, with the origin fixed and co‐moving with the tip of the strand (*x* = 0). b) Schematic shows the assumption of sequential progression of cell cycle used in the model. c) Model simulated distribution of cells in all phases in spheroids with only location‐dependent cell cycle entry. d) An intermediate forward advection speed of G2 cells is ideal for the model to reproduce experimentally observed cell cycle distribution along the strand. e) Spatiotemporal heatmaps show the dynamic distributions of proliferating Non‐G0, S, G2, and M cells after allowing G2 cells to migrate toward the tip in spheroids at a speed of 0.05 µm min^−1^. f) Model simulated distribution of cells in all phases in spheroids with both location‐dependent cell cycle entry and G2 cell forward motion. The insets on the right of (c) and (f) highlight the distribution of G2 and M cells.

We next developed a continuous RAD model to explore how the interplay between cell migration and proliferation may result in the seemingly contradictory distributions of S and M cells with opposite trends. The cells were assumed to migrate and proliferate along a 1D ray based on the quasi‐1D geometry of the strand near the tip (*x* = 0) (**Figure** **3**a). The fraction or percentage of each cell cycle phase at a given location *x* and time *t*, *ρ_i_* (*x*,*t*), is controlled by a set of 1D RAD equations as follows

(1) $$\frac{\partial \rho_{i}}{\partial t}=\frac{\partial }{\partial x}\left(D\frac{\partial \rho_{i}}{\partial x}\right)-\frac{\partial \left(V\rho_{i}\right)}{\partial x}+a_{i}e_{i}\rho_{i-1}-b_{i}e_{i+1}\rho_{i}+c\rho_{i}$$

where *i* = *G0*, *G1*, *S*, *G2*, or *M* representing each sequential cell cycle phase for simplicity (Figure 3b), *D* is the diffusion coefficient describing random cell migration, *V* is the advection speed describing directed cell migration, *e_i_* is the cell cycle phase transition rate, *a_i_/b_i_* = 1 or 2 account for source term factors due to cell cycle progression including cell division, and *c* describes additional source factors including apoptosis (see Experimental Section, Mathematical Modeling and Computer Simulation, for details).

Computer simulation of this RAD model with experimentally derived parameters (Table S1, Supporting Information) reproduced the distribution of S phase and proliferating cells but not M phase cells along invading strands (Figure 3c; Figure S4a, Supporting Information) assuming the cell cycle entry rate or G0‐to‐G1 transition rate increases with the distance to strand tip, *x*, following an error function,

(2) $$e_{G1}\left(x\right)=\frac{c_{1}erf\left(x/c_{2}\right)+1}{c_{1}+1}c_{3}$$

As cells in the G2 gap phase have a high energy level (Figure 1c) which often correlates with a high migration potential,^[^ ^8^ ^]^ we hypothesized that a forward‐directed movement of G2 cells may enable a shift in M phase distribution toward the front. To test this hypothesis, Equation (1) was modified to include an additional advection term, *V_G2_*, such that

(3) $$\begin{array}{ccc} \frac{\partial \rho_{G2}}{\partial t} & = & \frac{\partial }{\partial x}\left(D\frac{\partial \rho_{G2}}{\partial x}\right)-\frac{\partial \left(\left(V+V_{G2}\right)\rho_{G2}\right)}{\partial x}\\ & & +\;e_{G2}\rho_{G1}-e_{M}\rho_{G2}+c\rho_{G2} \end{array}$$

A forward advection speed of −*V_G2_* = 0.05 µm min^−1^ was sufficient to shift the distribution of M cells from rear‐concentrated to front‐concentrated within 30 min in the spheroid simulation (Figure 3d,e) and make the final distribution of all simulated cell cycle phases (Figure 3f) resemble that observed in experiments (Figure 2d). Moreover, the forward G2 advection alone seems to be sufficient to induce a distribution that qualitatively recapitulates experimental observation in spheroids (Figure S5a, Supporting Information) without the requirement of a location‐dependent cell cycle entry rate given by Equation (2). However, G2 advection alone will significantly concentrate M cells in the front in the organoid simulation, which was not observed in experiments, suggesting that a location‐dependent cell cycle entry is still required (Figure S4b–d, Supporting Information). Other than migration speed and location‐dependent cell cycle entry (Figure 3d; Figure S5a,b, Supporting Information), the model output is robust against most other parameters within a reasonable range, including cell doubling time, apoptosis, and initial cell cycle distribution (Figure S5c–e, Supporting Information). In sum, the RAD model we developed supports the hypothesis that a forward motion of G2 cells may be responsible for the observed proliferation profile along invading strands.

### 2.4 Cancer Cells Actively Move Toward the Invasion Front Before Cell Division

**Figure 4:**
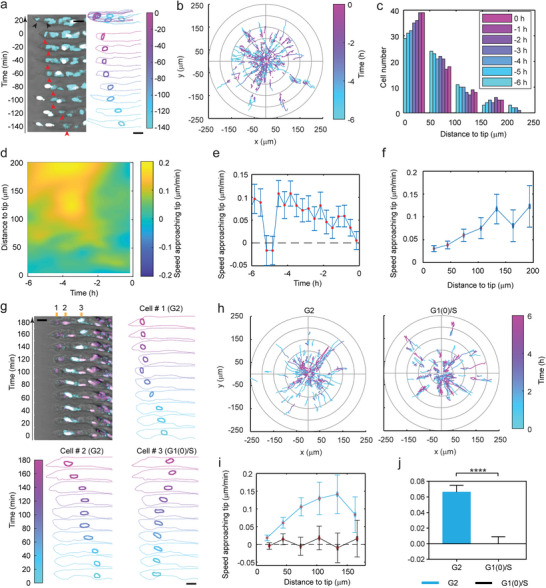
Cells migrate toward the tip of the strand before division. a) A representative time‐series image of MDA‐MB‐231 cells expressing the CycleTrak probe (white indicates G1(0) cells, cyan indicates S/G2/M cells), where a cell (indicated by red arrowheads) is migrating toward the tip of the strand before dividing at time t = 0 (black arrowheads indicate the two daughter cells after division). The outline of the strand and the cell nucleus of interest are color‐coded on the right. The tip of the strand is used to align the outlines to demonstrate cell migration relative to the tip (top right). b) The migration trajectories relative to the tip (*x* = 0, *y* = 0) color‐coded with the time before cell division (*t* = 0) demonstrate that most cells move closer to the tip before division (N = 163 tracked cells). c) The shifting of cell distribution as a function of time before division (*t* = 0) from a total of 70 tracked cells. d) A spatiotemporal heatmap shows the distribution of cell migration speed approaching the tip of the strand with regard to the distance to the tip and the time before cell division (*t* = 0). The relative migration speed of cells approaching the tip of the strand as a function of e) time before cell division (*t* = 0) and f, i) the distance to tip. g) A representative time‐series image of MDA‐MB‐231 cells expressing the Fucci4 probe shows the migration of G2 (magenta) and G1(0)/S (white) cells. The outline of the strand and the cell nuclei of interest are color‐coded with time for three typical cells (Cells #1, #2, #3). h) The migration trajectories relative to the tip (*x* = 0, *y* = 0) color‐coded with time demonstrate that most G2 cells move closer to the tip over time (N = 103 tracked cells), whereas G1(0)/S can move in either direction (N = 73 tracked cells). j) G2 cells approach the tip with a positive average speed whereas G1(0)/S cells do not (N = 1577, and 1425 speed data points, respectively). Data are pooled from at least three independent experiments. Error bar represents S.E.M. Scale bar, 25 µm. Statistical significance is tested by the Mann–Whitney test. **** *p* < 0.0001.

To test the model prediction of forward G2 cell motion, we next tracked the migration of MDA‐MB‐231/CycleTrak cells along invading strands and confirmed that cells tend to move toward the strand tip until the incidence of cell division (**Figure** **4**a,b, Video S3, Supporting Information). This forward migration progressively shifted the distribution of pre‐division cells toward the invasion front (Figure 4c). A close look at the spatiotemporal distribution of cell migration speed relative to the strand tip (Figure 4d; Figure S6a, Supporting Information) revealed that within ≈4 h before cell division, cells migrated at an average rate toward the tip (Figure 4e). By contrast, more than 4 h before cell division, the relative migration direction was more arbitrary (Figure 4e; Figure S6b, Supporting Information). This time scale corresponds to the transition of eukaryotic cells from the S phase to the G2 phase.^[^ ^30^ ^]^ The average speed of cells approaching the tip decreases when a cell approaches the time of division (Figure 4e) or approaches the tip position (Figure 4f; Figure S6c, Supporting Information), which is consistent with the fact that dividing cells are usually stationary and cells cannot move beyond the tip of the strand. Importantly, this average speed agrees well with the 0.05 µm min^−1^ value used in the previous model simulation (Figure 4e,f), further supporting a forward G2 motion hypothesis. The above analysis may incorporate some error, however, if the G2 phase duration spans a wide range. Hence, we next directly tracked the migration of G2 cells using the Fucci4 probe which labels G1(0)/S cells against G2/M cells via sequences from human stem‐loop binding protein (SLBP)^[^ ^28^ ^]^ (Figure 4g; Video S4, Supporting Information). The transient M phase was not included in the analysis as M cells are usually non‐migratory. Consistent with the migration before cell division, most G2 cells tend to move toward the tip, whereas the migration direction is more arbitrary for G1(0)/S cells with an average speed approaching the tip close to zero (Figure 4h–j; Figure S6d,e, Supporting Information), even though the magnitudes of their migration speeds are similar (Figure S6f, Supporting Information). Together, our results confirmed that G2 cells actively move toward the invasion front before cell division.

### 2.5 Enrichment of G2 Cells at the Leader Position Due to Forward Movement

**Figure 5:**
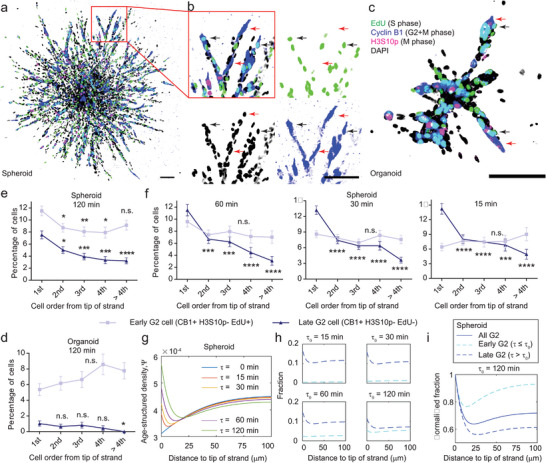
Accumulation of G2 cells at the invasion front due to forward streaming. Representative images of a) a tumor spheroid and c) an organoid invading collagen with respective nuclear markers for proliferation. b, c) EdU and cyclin B1 positive cells (CB1+ EdU+) are denoted as early G2 cells (black arrows), whereas cells stained positive for cyclin B1 but not for EdU (CB1+ EdU−) are denoted as late G2 cells (red arrows). N = 1580, 1514, 1228, 897, and 1247 cells for each cell order group from 22 spheroids, and N = 876, 762, 601, 454, and 656 cells for each cell order group from 174 organoids, pooled from at least two independent experiments, respectively. d) In organoids, early G2 cells are enriched at follower positions, whereas late G2 cells are enriched at the leader position. e, f) In spheroids, late G2 cells are always enriched at the leader position, whereas early G2 cells switch from a rear‐concentrated distribution to a front‐concentrated distribution with the increase of the EdU incubation period. N > 400 (1027, 899, 658, 424, and 465; 1650, 1574, 1236, 893, and 1068; 1157, 1123, 882, 562, and 584) cells for each cell order group from 20, 38, and 28 spheroids for the 15, 30, and 60 min EdU incubation conditions, respectively, which are pooled from at least two independent experiments. g) With the increase of G2 age (*τ*), simulated G2 cells at that age shift from a rear‐concentrated distribution to a front‐concentrated distribution. h, i) G2 cell forward motion with age‐structured simulation recapitulates the experimentally observed dependence of early G2 cell distribution on EdU incubation time (*τ*_0_) in spheroids. Statistical significance is tested by the Chi‐square test for independence, followed by multiple comparisons to the leader (first) group. Significances for multiple pair‐wise comparisons are adjusted with the Bonferroni correction. Error bar represents S.E.M. Scale bar, 100 µm. * *p* < 0.05, ** *p* < 0.01, *** *p* < 0.001, **** *p* < 0.0001, n.s.—not significant.

To further test the model prediction that the forward motion of G2 cells leads to their frontal enrichment, we next stained the spheroids and organoids with cyclin B1 (**Figure** **5**a–c). Cyclin B1 is actively expressed and accumulated at the cytoplasm mainly in the G2 phase (Figure S7a–c, Supporting Information) and degrades during the M phase.^[^ ^31^ ^]^ Hence, the combination of cyclin B1 and a M phase marker identifies G2 cells. Consistent with the model prediction (Figure 3f), G2 cells were highly enriched at the leader position with a slope steeper than that of M cells in MDA‐MB‐231 spheroids (Figure S7d, Supporting Information), supporting the idea that frontal enrichment of G2 cells causes the increased cell division at the tip. Similar observations were confirmed in MDA‐MB‐231 spheroids in collagen matrices of different densities and in NIH3T3 spheroids, as well as in MDA‐MB‐231 spheroids expressing the Fucci4 probe (Figures S2e–h and S3h, Supporting Information). Not out of expectation, we did not observe a significant frontal enrichment of G2 cells for MMTV‐PyMT organoids and 4T1 spheroids since in these systems neither S nor M cells were front‐concentrated (Figures S3h and S7e, Supporting Information). However, G2 cells appear to be front‐concentrated in all spheroids and organoids when quantifying the relative percentage against S cells (Figures S3i and S7f,g, Supporting Information), supporting the hypothesis that G2 cells migrate forward relative to S cells in all 3D streaming systems we tested. More interestingly, after separating G2 cells into two groups, i.e., “early” and “late” G2 cells (Figure 5b,c), we found late G2 cells in organoids were front‐concentrated, whereas early G2 cells were rear‐concentrated (Figure 5d; Figure S8a, Supporting Information). Here, early G2 cells are defined as cells with a G2 age, *τ* (defined as the time a cell has spent in the G2 phase), equal or smaller than *τ*_0_ (determined by the EdU incubation period in experiments, see Experimental Section for details). Hence, the above results suggest that the longer time of forward migration allowed late G2 cells to accumulate at the leader position in organoids (Figure 5d; Figure S8b, Supporting Information), even though G2 cells as a whole were not front‐concentrated (Figure S7e, Supporting Information). Similarly, late G2 cells appeared to be more concentrated at the leader position than early G2 cells in MDA‐MB‐231, NIH3T3, and 4T1 spheroids (Figure 5e,f; Figure S8c, Supporting Information). However, early G2 cells in MDA‐MB‐231 spheroids also appeared to be front‐concentrated (Figure 5e; Figure S8c, Supporting Information), which may be caused by the shorter time it takes for G2 cells to move and enrich at the tip in MDA‐MB‐231 spheroids than in MMTV‐PyMT organoids. To test this hypothesis, we progressively reduced the EdU incubation period from 120 to 15 min for MDA‐MB‐231 spheroids, thus reducing the average age of early G2 cells. As expected, the percentage of early G2 cells but not the overall distribution of S, G2, and M cells decreased with decreasing EdU incubation period (Figure 5e,f; Figure S8d,e, Supporting Information). Moreover, the early G2 distribution curve became progressively flatter and eventually flipped from a negative slope to a positive slope (Figure 5e,f; Figure S8f–h, Supporting Information).

To test whether the above explanation can be reproduced using our RAD model, Equation (3) was modified to include the age parameter, and the control equations for G2 cells now read:

(4) $$\begin{array}{c} \left\{\begin{array}{c} \frac{\partial \psi_{G2}}{\partial t}+\frac{\partial \psi_{G2}}{\partial \tau }=\frac{\partial }{\partial x}\left(D\frac{\partial \psi_{G2}}{\partial x}\right)-\frac{\partial \left(\left(V+V_{G2}\right)\psi_{G2}\right)}{\partial x}-{e_{M}}^{\prime }\psi_{G2}\left(\tau >\tau^{\prime }\right)+c\psi_{G2}\\ \psi_{G2}\left(x,t,0\right)=e_{G2}\rho_{S}\left(x,t\right)\\ \rho_{G2}\left(x,t\right)=\int_{\tau \geq 0}\psi_{G2}\left(x,t,\tau \right)d\tau \end{array}\right.\;\;\;\;\;\; \end{array}$$

where ψ_*G*2_(*x*,*t*, τ) is the fraction of G2 cells with an age of *τ*, τ′ is a refractory period within which G2 cells will not transition to the M phase,^[^ ^32^ ^]^ and *e_M_* ′ is the G2‐to‐M transition rate for non‐refractory G2 cells. Computer simulation clearly demonstrated the shift of age‐structured G2 distribution toward the front with the progression of time (Figure 5g) and reproduced the distribution of early and late G2 cells with a similar trend as observed in spheroid experiments for different *τ*_0_ (Figure 5h,i; Figure S5g–i, Supporting Information). The model also reproduced the distributions for early and late G2 cells in organoids (Figure S4e, Supporting Information). Reducing the slope of the location‐dependent cell cycle entry rate and increasing the G2 forward migration speed made early G2 cells in organoids more concentrated toward the tip (Figure S4f–h, Supporting Information), suggesting that these parameters may contribute to the difference in G2 distribution observed between spheroids and organoids. Taken together, our results demonstrated that when the cells just enter the G2 phase, their distribution is affected by and follows that of S cells, and that with the progression of time, G2 cells preferentially migrate and progressively shift the overall distribution toward the front.

### 2.6 Forward Motion of G2 Cells is Driven by Their High Energy Level

**Figure 6:**
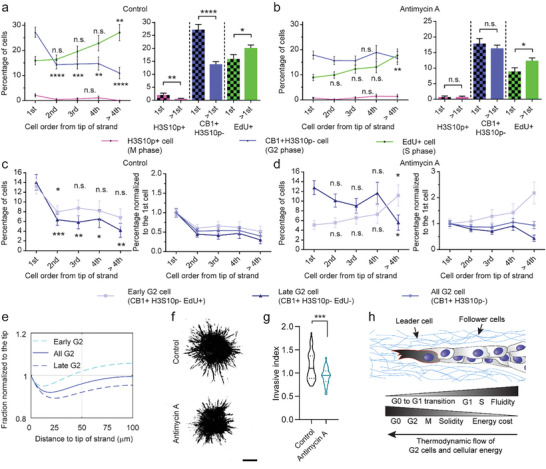
High energy level drives G2 cell forward motion to support cancer invasion. Compared to a) control spheroids, b) reducing the high energy level in G2 cells by antimycin A treatment eliminates the difference between leader and follower positions in G2 and M cells, but not in S cells. N = 477, 439, 307, 184, and 192 cells for each cell order group from 32 control spheroids, and N = 548, 504, 365, 206, and 215 cells from 33 treated spheroids, respectively, which are pooled from three independent experiments. d) With antimycin A treatment, early G2 cells follow closely with the distribution of S cells, suggesting diminished forward motion of the cells after they enter the G2 phase, which is different from c) control. e) Computational simulation using 20% of G2 forward migration speed recapitulates the trend of early and late G2 cell distribution observed with antimycin A treatment. f) Representative images of MDA‐MB‐231 spheroids 48 h post embedding in 4.5 mg mL^−1^ collagen showing short and wide strands with 24 h antimycin A treatment. g) Antimycin A treatment reduces the invasive migration of spheroids into surrounding matrices (N = 40, and 33 spheroids pooled from three independent experiments, respectively). h) A biophysical model of collective dynamics in invading strands. Statistical significance is tested by Student's *t*‐test or Chi‐square test for independence, followed by multiple comparisons to the leader (first) group. Significances for multiple pair‐wise comparisons are adjusted with the Bonferroni correction. Error bar represents S.E.M. Scale bar, 200 µm. * *p* < 0.05, ** *p* < 0.01, *** *p* < 0.001, **** *p* < 0.0001, n.s. – not significant.

Given that the energy level in cancer cells correlates with their migratory potential in 3D^[^ ^33^ ^]^ and leader cells consume more energy than follower cells,^[^ ^8^ ^]^ we hypothesized that the pro‐migratory phenotype of G2 cells is attributed to their high energetics (Figure 1c). To test this hypothesis, we treated MDA‐MB‐231 spheroids with antimycin A to eliminate the energetic advantage of G2 cells over other cells and found it eliminated the difference between leader position and follower positions in G2 and M phase distributions (**Figure** **6**a,b). The effect of antimycin A treatment was most obvious in the distribution of early G2 cells, which became similar to that of S cells (Figure 6c,d). The observation that late G2 cells were still slightly concentrated toward the leader position suggests that the decreased energy production by antimycin A treatment may have slowed down instead of completely aborted the forward motion of G2 cells. This effect of slowing down can be recapitulated in our model simulation by simply reducing the advection speed by 80–90% (Figure 6e; Figure S5j–l, Supporting Information). However, we cannot exclude the possibility that an energy‐independent mechanism may also contribute to the frontal enrichment of late G2 cells. Interestingly, antimycin A did not affect the rear enrichment of S cells (Figure 6a,b), suggesting that the location‐dependent cell cycle entry is independent of cellular energetics or G2 advection. In contrast to antimycin A, 2‐DG, which was not able to suppress the energy cycle (Figure S1b, Supporting Information), failed to stop the frontal enrichment of G2 cells (Figure S9a, Supporting Information). Moreover, while both antimycin A and 2‐DG significantly inhibited the collective invasion of tumor spheroids into the surrounding matrices (Figure 6f,g; Figure S9b–d, Supporting Information) likely due to their inhibitory effects on cell cycle progression (Figure S1c,d, Supporting Information), they have different impacts on the morphology of the invading strands. Invading strands with 2‐DG treatment assumed a morphology with a thin width and a sharp tip, similar to that of control (Figure S9b, Supporting Information). By contrast, invading strands with antimycin A treatment assumed a morphology with a shortened length and widened width (Figure S9c, Supporting Information), a sign indicating that the strands are expanding in volume but may have difficulty invading forward. In addition, MDA‐MB‐231 cells containing external mitochondria (exogenous mitochondria extracted from other cells, see the Experimental Section for more details), which were previously shown to exhibit increased energy levels and stay at the leader position for elongated durations,^[^ ^8^ ^]^ moved toward the tip whereas control cells moved or were pushed away from the tip on average when both types of cells were present in a hybrid strand (Figure S9e,f, Video S5, Supporting Information). While the average speed here is small compared to the average speed of G2 cells alone, the highly significant difference further supports the hypothesis that high‐energy production from mitochondria powers the forward motion in strands. Together, our data suggest that cell cycle‐mediated high energy production in G2 cells enables them to obtain a pro‐migratory phenotype, which drives the forward tumor invasion (Figure 6h).

## 3 Discussion

Our study clarified the long‐standing problem of “go or grow” by giving insights into the bioenergetics that connect proliferation and migration and uncovered an advective flow of cells and energy that may be fundamental to 3D collective dynamics. How is this cellular flow possible? From a thermodynamic perspective, healthy tissues are active matter that self‐organizes in a way that deviates from equilibrium, whereas cancer progression reverses this trend and moves toward the direction of maximum disorder or entropy. As system entropy is often positively correlated with the total energy of the system, it is not out of expectation that high‐energy G2 cells have greater potential to become disorganized and present in the invasion front as compared to cells in the low‐energy phases (Figure S7, Supporting Information). In addition, our previous work demonstrated that leader cells consume more energy than follower cells during collective migration in 3D,^[^ ^8^ ^]^ suggesting that there may exist an energy barrier preventing low‐energy cells from entering the frontal region. Supporting this idea, we observed a decrease in random migration speed toward the tip and the gradient extends for more than 100 µm from the tip before the speed plateaus (Figure S6f, Supporting Information). Cell migration toward this 100‐µm frontal region is difficult and consumes additional energy such that higher‐energy cells are more likely to enter this region. Part of this additional energy may be consumed to deform the stiff environment at the front, as within the invading strands the frontal region can be stiffer and more solid‐like than the rear region,^[^ ^34^ ^]^ likely due to the ability of frontal cancer cells to remodel the matrix which increases the stiffnesses of both the cell and the matrix.^[^ ^35^ ^]^ This locally increased solidity toward the invasion front is opposite to the general solid‐to‐fluid transition trend during cancer invasion where invading strands are often softer than the center of the spheroids,^[^ ^7^ ^]^ thus it deserves further investigation.

While we confirmed G2 cells, but not G0/G1/S cells migrate at an average speed toward the tip (Figure 4), the instantaneous migration direction of individual cells appears arbitrary and the speed magnitude is much larger than that of the average speed^[^ ^8^ ^]^ (Figure S6, Supporting Information). As a result, the forward streaming of G2 cells can only be captured at the collective level described by statistical physics. The decoupling of cellular motion at the individual level in the strands implies a high degree of disorder and fluidity during cancer invasion, consistent with previous work.^[^ ^7^ ^]^ Hence, we developed a 1D RAD model to describe this continuous fluid‐like collective behavior. Our model implies that cell migration speed and location‐dependent cell cycle entry are the most important factors contributing to the distribution of cell cycle phases along invading strands. Considering the different cell‐ECM interactions between leader and follower positions,^[^ ^8^ ^]^ and that mechanical stretch or strain often regulates cell cycle entry,^[^ ^36^ ^]^ we speculate that the differential mechanical forces experienced by the leader and follower cells may contribute to this location‐dependent cell cycle entry. A location‐dependent rear enrichment of S phase cells appears to be a robust observation in all 3D collective streaming systems tested (Figure 2d; Figure S3e,h, Supporting Information). However, likely due to a different microenvironmental setting, this is not often observed and can be just the opposite for 2D monolayers^[^ ^37^ ^]^ (Figure S3k, Supporting Information). In addition, the differential mechanical microenvironment may also contribute to the increased cell division at the leader position, since mechanical stretch, wounding, and epithelial expansion have been shown to trigger cell division.^[^ ^3^, ^38^ ^]^ While the RAD model also suggests that a speed profile for G2 cells resembling that of experimental measurements generates better results than a simple constant speed profile (Figure S5, Supporting Information), we did not intend to build all the details into the model, especially since many experimental variables are still not known. For example, unlike their 2D counterparts,^[^ ^10^ ^]^ it remains challenging to measure and estimate intercellular stresses and cell–matrix and cell–cell frictions in 3D strands to develop a similar model that includes force balance, constitutive equations, and the mechanical feedback to cell cycle progression.^[^ ^39^, ^40^ ^]^ Instead, we used the RAD equations based on conservation laws and experimentally derived parameters (Table S1, Supporting Information) that are closely connected to the 3D microenvironment to help understand the associated biophysical principles. Given our experimental and modeling outcomes, the forward motion of G2 cells appears to be a robust feature of the 3D collective stream considering the consistent front‐enriched G2/S ratio distributions (Figures S3i and S7f,g, Supporting Information), whereas G2 cells by themselves may or may not be enriched to the front (Figures S3h and S7d,e, Supporting Information), which depends on multiple factors including the S phase distribution, the G2 phase migration speed and the G2 phase duration (Figures S3h, S4e–h, and S7d,e, Supporting Information).

Using the fluorescent ubiquitination‐based cell cycle indicator (FUCCI)^[^ ^42^ ^]^ and its derivatives,^[^ ^29^ ^]^ live cells in the G0/G1 phase and S/G2/M phase can be identified and are often categorized as nonproliferative and proliferative cells, respectively.^[^ ^18^, ^21^ ^]^ Our experimental and modeling results suggest that critical cell cycle‐dependent information may be lost with this simple two‐state categorization. For example, we showed that in MDA‐MB‐231 cells, G0 and G1 phases, and S and G2/M phases, have opposite trends in their distributions, such that the ensemble distribution of G0/G1 or S/G2/M phases may be ambiguous and dictated by the cell cycle phase with more cells (Figure 3f; Figures S2h and S9a, Supporting Information). Recent work using the FUCCI probe found that invadopodia activity, which is important for cancer cell migration in 3D, is enhanced in the G0/G1 phase and may be responsible for leader cell invasion.^[^ ^43^ ^]^ It is possible, however, that invadopodia are enhanced in the G0 instead of the G1 phase, considering the frontal enrichment of G0 but not G1 cells. Due to their quiescent state, cellular energy, albeit at a low level, may be conserved from the proliferation machinery to support invasion at the tip in the G0 phase. In the same study, G0/G1 phase leader cells were found to migrate backward once they entered the S phase, which is consistent with our data showing S phase cells are rear‐concentrated (Figure 2). It is likely that most of the cellular energy is directed to DNA synthesis and not readily available for cytoskeletal activities in S‐phase cells such that forward migration is prohibited despite their relatively high energy level. Unlike S‐phase cells, the high energy level and low biosynthesis activity in the G2 gap phase may allow G2 cells to move toward the more energy‐demanding leader position,^[^ ^8^ ^]^ which then drives the invasion of the strand. Hence, it is of critical importance to specifically investigate cell behaviors in each cell cycle phase using advanced probes,^[^ ^28^, ^44^ ^]^ especially for the front‐concentrated G2 and G0 phase cells. We used EdU and cyclin B1 double staining as a means to track the age of G2 cells. However, since cyclin B1 synthesis starts at the S phase^[^ ^30^ ^]^ albeit at a very low level^[^ ^31^ ^]^ (Figure S7a–c, Supporting Information), some of the detected early G2 cells may actually be late S cells using this method. Hence, it is possible that late S cells also flow toward the front due to their increased energy production compared to G0/G1 cells. Nevertheless, the data agrees with the hypothesis that differentially regulated cellular energetics drive the forward streaming of cancer cells, which was confirmed by tracking the motion of cells before division and cells expressing the Fucci4 probe that specifically detects G2 cells.

ATP energy is required by the molecular players in both the proliferation^[^ ^22^ ^]^ and the migration^[^ ^12^ ^]^ machinery, such that high ATP availability may promote both processes whereas limited ATP availability may lead to the “go or grow” competition. While cancer cells may rely on different pathways to produce energy,^[^ ^12^ ^]^ our results suggest that among the two major ATP production pathways, mitochondrial oxidative phosphorylation may be more important than glycolysis to support an ATP energy cycle. Migration of cells in 3D is more difficult than that in 2D as cells in 3D often need to degrade or deform the surrounding matrices or deform themselves to move forward.^[^ ^45^ ^]^ All of these 3D cell–matrix interactions consume energy, such that 3D cell migration is more sensitive to energy availability and fluctuations,^[^ ^46^ ^]^ and thus more likely to be affected by the energy cycle compared to 2D cell migration.^[^ ^21^ ^]^ Indeed, we found that a front‐to‐rear differential distribution of cell cycle phases is less obvious at the 2D wound edge as compared to invading 3D strands, such that the distribution of G2/M phase cells often follows that of S phase cells, regardless of if S phase is front‐/rear‐concentrated or not (Figure S3k,l, Supporting Information). However, other factors like mechanical stretch,^[^ ^38^ ^]^ lateral confinement, and cell density^[^ ^3^ ^]^ may directly trigger cell cycle entry or create a front‐to‐rear energy barrier and still contribute to increased frontal G2 cell presence in 2D under certain conditions. Nevertheless, this cell cycle‐regulated energy variation may have an impact on various other energy‐consuming processes in both 2D and 3D such as cell contractility and epithelial remodeling,^[^ ^47^ ^]^ in addition to migration as we observed in this study. While nutrient and oxygen availability may also contribute to increased cell division as seen in the periphery versus the core in spheroids,^[^ ^48^ ^]^ a gradient of nutrient or oxygen is not likely to exist for the thin strand structures.^[^ ^8^ ^]^ We previously suggested that in a more heterogeneous cell population, tip cells of a leader phenotype (for example, cells expressing keratin‐14 in MMTV‐PyMT organoids^[^ ^5^ ^]^ and cells with elevated mitochondrial respiration in lung cancer cells^[^ ^41^ ^]^) are less likely to be replaced by cells following them unless they are also of a leader phenotype.^[^ ^8^ ^]^ Similarly, it might be difficult for a high‐energy G2 cell of a non‐leader phenotype to move forward and surpass a lower‐energy G0/G1 cell of a leader phenotype if the phenotypic difference is big enough. As a result, there might not be enough G2 cells enriched toward the front even though G2 cells do tend to move forward compared to G0/G1/S cells of the same phenotype, and this may also help explain why no frontal enrichment of G2 cells was observed in our organoid system (Figure S7e, Supporting Information). Nevertheless, as long as G2 cells do move forward relative to G0/G1/S phase cells of the same phenotype, the G2/S ratio will be distributed toward the front, and this was indeed consistently observed in all of our spheroids and organoid systems (Figures S3i and S7f,g, Supporting Information).

In sum, using principles from statistical physics and thermodynamics coupled with experiments, we demonstrated that cell cycle progression mediates a corresponding bioenergetic cycle, which feedbacks to regulate cancer cell migration, and G2 cells may be the most devastating among cells in all phases due to their high energy level and pro‐migratory phenotype. This knowledge may help identify therapeutic interventions to target the most aggressive cells and disrupt the reciprocal feedback between proliferation and migration that contributes to cancer metastasis. Furthermore, we described a new type of bioenergetics‐based morphogenetic flow, which may be fundamental to collective dynamics in 3D. Bioenergetic fluctuation that arises from cell cycle progression or intercellular heterogeneity may be sufficient to induce a generic morphogenetic flow toward an energy barrier gradient. Without such an energy barrier, the morphogenetic flow may still exist but may become less obvious, such as in the 2D wound healing case (Figure S3j–l, Supporting Information).

## 4 Experimental Section

### Cell Culture, Plasmids, and Reagents

MDA‐MB‐231 human breast adenocarcinoma cells (Catalog # HTB‐26, ATCC) and NIH3T3 mouse fibroblasts (Catalog # CRL‐1658, ATCC) were maintained in Dulbecco's Modified Eagle Medium (DMEM) with high glucose (4.5 g L^−1^; Catalog # 11965092, Life Technologies). 4T1 mouse mammary carcinoma cells (Catalog # CRL‐2539) were maintained in RPMI 1640 (ATCC modification; Catalog # A1049101, Life Technologies). Breast cancer‐associated fibroblasts (CAFs) were kindly provided by Dr. Andra Frost (University of Alabama at Birmingham, Birmingham, AL) and were maintained in DMEM with low glucose (1.0 g L^−1^; Catalog # 11885084, Life Technologies). All cell culture media were supplemented with 10% fetal bovine serum (FBS; Atlanta Biologicals), 100 µg mL^−1^ streptomycin (Life Technologies), and 100 U mL^−1^ penicillin (Life Technologies). All cell culture and live cell imaging were performed in a humidified environment at 37 °C and 5% CO_2_. The cell lines used were tested for mycoplasma and deemed free of contamination.

The CycleTrak^[^ ^29^ ^]^ plasmid (Fucci‐Orange‐G1/2A/H2B‐eGFP) was kindly provided by Dr. Paul M. Kulesa (Stowers Institute for Medical Research, Kansas City, MO), and was expressed in MDA‐MB‐231 cells via lentiviral transduction as described previously.^[^ ^8^, ^29^ ^]^ The two Fucci4^[^ ^28^ ^]^ probe plasmids, Clover‐Geminin(1‐110)‐IRES‐mKO2‐Ctd(30‐120) (Addgene plasmid #83841; denoted as Geminin/Ctd plasmid hereafter) and mTurquoise2‐SLBP(18‐126)‐IRES‐H1‐mMaroon1 (Addgene plasmid #83842; denoted as SLBP/H1 plasmid hereafter), were gifts from Dr. Michael Lin (Stanford University, Stanford, CA), and were coexpressed in the cell line through lentiviral transduction. Briefly, Geminin/Ctd and SLBP/H1 lentiviral particles were prepared by transient transfection of HEK293T cells with the lentiviral expression vectors and the second‐generation packing constructs psPAX2 and pMD2.G in the presence of TransIT‐LT1 (Catalog# MIR2300, Mirus). Lentiviral particles were harvested from the HEK293T supernatant at 48 and 72 h post‐transfection and concentrated with the Lenti‐X Concentrator (Catalog# 631231, Clontech) followed by stable MDA‐MB‐231 cell transduction in the presence of 8 µg mL^−1^ polybrene (Catalog# sc‐134220, Santa Cruz Biotechnology) overnight. To select for cells expressing both Fucci4 plasmids, the cells were sorted for both mTurquoise2 and mKO2 signals using a BD FACSAria lll Cell Sorter at the Vanderbilt University Medical Center (VUMC) Flow Cytometry Shared Resource Core. GW1‐pHRed (Addgene plasmid #31473) and FUGW‐PercevalHR (Addgene plasmid #49083) were gifts from Dr. Gary Yellen (Harvard Medical School, Boston, MA), and were coexpressed in MDA‐MB‐231 cells via lentiviral transduction as previously described.^[^ ^33^ ^]^

Cells were treated with 20 µm antimycin A (Catalog # A8674, Sigma) for 24 h to inhibit mitochondrial respiration by inhibiting mitochondrial electron transport chain complex III as previously described.^[^ ^46^ ^]^ Cells were treated with 25 mm 2‐deoxy‐d‐glucose (2‐DG, Catalog # D6134‐1G, Sigma) for 24 h to inhibit glycolysis as previously described.^[^ ^49^ ^]^

### Spheroid and Organoid Preparation

MDA‐MB‐231, NIH3T3, and 4T1 spheroids were generated as previously described.^[^ ^6^, ^8^ ^]^ Briefly, cells were harvested at ≈80% confluency and resuspended in DMEM/F12 (Life Technologies), supplemented with 0.25% methylcellulose (Catalog # 04100, Stem Cell Technologies), 4.5% horse serum (Catalog # 16050130, Life Technologies), 18 ng mL^−1^ hEGF (Catalog # PHG0311, Life Technologies), 0.45 µg mL^−1^ hydrocortisone (Catalog # H0888, Sigma), 9 µg mL^−1^ insulin (Catalog # I6634, Sigma), 90 ng mL^−1^ cholera toxin (Catalog # C8052, Sigma), 90 U mL^−1^ penicillin, and 90 µg mL^−1^ streptomycin, or resuspended in the respective cell culture medium, supplemented with 0.25% methylcellulose. The cell suspension was then seeded at 5000 cells per well to a 96‐well round‐bottom microplate, centrifuged at 300 × g for 5 min, and incubated for 2–3 days for spheroid to form.

Tumor organoids were generated from 12–14‐week‐old MMTV‐PyMT mice, which were maintained following a protocol approved by the Vanderbilt University Institutional Animal Care and Use Committee (Protocol number M1700029, Animal Welfare Assurance A3227‐01), as described previously.^[^ ^8^ ^]^ Briefly, mammary tumor tissues harvested from the mice were minced with a pair of scalpels and then digested into small fragments at 37 °C for 30–50 min in DMEM/F12 supplemented with 0.2% collagenase (Catalog # 17104019, Life Technologies), 0.2% trypsin (Life Technologies), 5 µg mL^−1^ insulin, 50 µg mL^−1^ gentamicin (Catalog # 15750060, Life Technologies) and 5% FBS. The digested tissues were collected by centrifuging at 500 × g, treated with 2 U µL^−1^ DNase (Catalog # M0303L, New England BioLabs), and resuspended in DMEM/F12. Differential centrifugation was then applied to isolate organoids from single cells. DMEM/F12, supplemented with 100 U mL^−1^ penicillin and 100 µg mL^−1^ streptomycin, 1% insulin–transferrin–selenium (Catalog # I3146, Sigma), and 2.5 nm bFGF (Catalog # PHG0026, Life Technologies) was used as organoid culture medium.

Compacted spheroids or extracted organoids were resuspended in acid‐solubilized type I rat tail collagen stock solution diluted to 4.5 mg mL^−1^ in complete culture medium unless otherwise stated. To induce collagen polymerization, 1 n NaOH was used to neutralize the collagen solution to pH 7.0, which was pipetted immediately to glass‐bottom microplates (MatTek Corporation) together with the desired number of spheroids/organoids and incubated at 37 °C for 30 min. The polymerized collagen gels were then overlayed with the respective cell culture media, and the embedded spheroids/organoids were allowed to invade for 2–3 days before fixation or live‐cell imaging.

### Microscopy

Microscopic images were taken with a Zeiss LSM800 or a Zeiss LSM900 confocal microscope, equipped with an environmental chamber. Time‐lapse images were taken every 20 min for up to 20 h unless otherwise stated. A 10× dry lens (N.A. = 0.3) was used to track cell nuclei within invading strands with 25‐µm‐interval Z‐stacks, to track the outgrowth of spheroids with 10‐µm‐interval Z‐stacks, and to image the distribution of cell proliferation in spheroids and organoids with 8‐µm‐interval Z‐stacks. A 20× dry lens (N.A. = 0.8) was used to monitor cellular ATP/ADP ratio with regard to cell cycle distribution with 5‐µm‐interval Z‐stacks and to track cell migration in spheroids expressing Fucci4 with 3 µm‐interval Z‐stacks. Confocal Z‐stack images of spheroids and organoids were shown as maximum intensity projections across all Z stacks unless otherwise stated.

### Measurement of Intracellular ATP/ADP Ratio and Tracking of Cell Cycle Progression

Intracellular ATP/ADP ratio was measured in MDA‐MB‐231 cells expressing the PercevalHR and pHRed probes, as described previously.^[^ ^33^, ^50^ ^]^ Briefly, the PercevalHR probe was excited with a 488‐ or 405‐nm laser, and emission light was collected over the 410–546 nm range, whereas the pHRed probe was excited with a 561‐ or 488‐nm laser, and emission light was collected over the 576–650 nm range, resulting in a total of four images taken roughly at the same time. Pixel‐by‐pixel PercevalHR and pHRed ratio images were then calculated from the two PercevalHR images and two pHRed images and denoted as *r_PercevalHR_* and *r_pHRed_*, respectively. As the PercevalHR sensor is sensitive to pH,^[^ ^50^ ^]^ a transient alkalization of the cytosol was induced by adding 20 mm NH_4_Cl to the cell culture medium to obtain a calibration curve between *r_PercevalHR_* and *r_pHRed_*, assuming that the cellular ATP/ADP ratio did not change during the 2–3 min short calibration period. The calibration data was then fitted with a linear equation, *r_PercevalHR_* = *kr_pHRed_* + *q*, with *k* and *q* being the slope and intercept, respectively. This linear equation was then used to correct the pH dependence of *r_PercevalHR_* in experiments, such that the corrected PercevalHR ratio became *r_corr_* = *r_PercevalHR_* − *kr_pHRed_*. The corrected PercevalHR ratio was normalized to the mean value of the control group unless otherwise stated and designated as the normalized ATP/ADP ratio, *r_norm_* = *r_corr_*/*mean*(*r_corr_* (*control*)).

To monitor cell cycle progression at the same time, individual cells seeded in glass‐bottom microplates were labeled with 2.5 µm of the far‐red nuclear dye DRAQ5 (Catalog # 62251, Thermo Scientific) in growth medium, which was excited with a 640‐nm laser and emission light was collected over 656–700 nm range. After incubating with DRAQ5 for 2–3 h to allow the fluorescent intensity to stabilize,^[^ ^24^ ^]^ cells were imaged for both ATP/ADP ratio and nuclear stain. An average projection image was created from each Z‐stack image, and from which each nucleus was segmented by thresholding using the ImageJ (National Institutes of Health) tool Auto Threshold with Otsu's method and identified using the ImageJ tool Analyze Particles with manual supervision. The integrated intensities of all the nuclei were then plotted as a histogram as previously described,^[^ ^23^, ^24^ ^]^ and the gating values for separating G0/G1, S, G2/M phases of the cell cycle were determined using the flow cytometry software FCS Express 7 (De Novo Software) with the Multicycle AV add‐on (Phoenix Flow Systems) using a 1‐cycle fit.^[^ ^51^ ^]^ Basically, Multicycle builds a mathematical model of the histogram by fitting the G0/G1, G2/M peaks as Gaussian curves and the S phase distribution as a Gaussian‐broadened distribution, from which the percentage of G0/G1, S, G2/M phases and the corresponding gating values can be determined.

Alternatively, MDA‐MB‐231 cells expressing the PercevalHR and pHRed probe were imaged as described above every 30 min for up to 60 h. Cells that completed one or two divisions were manually tracked before, between, and after cell divisions, and the corrected PercevalHR ratio was normalized to the ratio when the cell was at its first division and designated as the normalized ATP/ADP ratio, such that *r_norm_* = *r_corr_*/*r_corr_* (*t* = 1st divion). The time of cell division was defined as the last frame before a parent cell divided into two daughter cells. To get an average trend of cellular energy dynamics over consecutive divisions, for cells completed two cell divisions, the total duration between the two cell divisions was normalized to 1 and the normalized ATP/ADP ratios were interpolated onto a regular grid on the axis of this normalized cell cycle.

### Labeling of Cell Proliferation

Proliferation in spheroids and organoids was labeled using the Click‐iT Plus EdU Cell Proliferation Kit for Imaging, Alexa Fluor 488 dye (Catalog # C10637, Invitrogen), and antibody‐based proliferation markers. Briefly, spheroids and organoids were incubated with 10 µm 5‐ethynyl‐2′‐deoxyuridine (EdU), a nucleoside analog of thymidine, in growth medium for 2 h at 37 °C. For MDA‐MB‐231 spheroid experiments, EdU incubation time were varied from 15 min to 2 h to identify the shift in early G2 cell distribution. Following EdU incubation, cells were immediately fixed with 3.7% formaldehyde (Sigma) in phosphate‐buffered saline (PBS) and permeabilized with 1% Triton X‐100. To detect the EdU signal, the cells were incubated with the Click‐iT reaction cocktail according to the manufacturer's instruction for 2 h at room temperature. Following the EdU assay, cells were stained with primary antibodies against Ki‐67 (Catalog # PA5‐19462 or 14‐5698‐82, Invitrogen), Cyclin B1 (Catalog # 4138S, Cell Signaling Technology), phospho‐histone H3 (Ser10) (H3S10p; Catalog # 9701, Cell Signaling Technology; or Catalog # MABE939, EMD Millipore). Alexa Fluor 568 or Alexa Fluor 647 conjugated secondary antibodies (Catalog # A10042 and A21247, Invitrogen), and DAPI (Catalog # D1306, Invitrogen) were used to detect the signals from the primary antibodies and from the nuclei.

### Validation of Cyclin B1 as a Marker for G2 Cells

To validate the use of cyclin B1 immunofluorescent signal in the cytoplasm as a marker for G2 cells, MDA‐MB‐231 cells expressing the Fucci4 probe cultured on glass substrates were fixed with 3.7% formaldehyde, permeabilized with 1% Triton X‐100 and labeled with the primary antibody for cyclin B1 as described above. Following that, a reference image of the Fucci4 signal was taken by exciting mTurquoise2 with a 405‐nm laser, Clover with a 488‐nm laser, mKO2 with a 561‐nm laser, and mMaroon1 with a 640‐nm laser. The cells were then stained with the Alexa Fluor 647 conjugated secondary antibody against the primary cyclinB1 antibody. A second image of the Fucci4 and cyclin B1 signal was taken using the same imaging parameters. While mMaroon1 from the Fucci4 probe and cyclin B1 have overlapping fluorescent spectrum, due to the nuclear location of the Histone H1 construct (H1‐Maroon1),^[^ ^28^ ^]^ the nuclear mMarroon1 signal has little interference with the cytoplasmic cyclin B1 signal. The nuclear cyclin B1 signal can be further obtained by subtracting the mMaroon1 signal in the reference image from the mMaroon1/cyclin B1 signal in the second image.

Cell cycle stages were then determined from the Fucci4 probe signals. Briefly, the nuclear Fucci4 signals were used to segment the cell nuclei using the ImageJ tool Auto Threshold with Otsu's method after background subtraction. The cell nuclei were identified using the ImageJ tool Analyze Particles with manual supervision. The average nuclear signals for all four Fucci4 channels were measured for each identified cell nucleus and a histogram was generated for each Fucci4 signal. Otsu's method was used to determine a threshold value based on the histogram for each channel using MATLAB (MathWorks). According to Bajar et al.,^[^ ^28^ ^]^ mKO2‐ Cdt1_30‐120_ is expressed in G1(0)/early S phase, mTurquoise2‐SLBP_18‐126_ is expressed in G1(0)/S phase, Clover‐Geminin_1‐110_ is expressed in S/G2/M phase, whereas H1.0‐mMaroon1 becomes condensed and bright in the M phase but never completely disappears in any cell cycle phases. Hence, Otsu's threshold could be used to determine the expression of the Fucci4 channels except for the mMaroon1 channel. Cells with a nuclear intensity above the Otsu threshold were considered positive for the specific Fucci4 channel. To be conservative, cells with a nuclear intensity smaller than half of the Otsu threshold were considered negative for the specific Fucci4 channel, and any cells with nuclear intensity above half of the Otsu threshold but below the Otsu threshold were considered undetermined for the specific Fucci4 channel. For the mMaroon1 signal, because it never completely disappears in any cell cycle stage, any cells with mMaroon1 signal above half of the Otsu's threshold were considered to be positively expressing the mTurquoise2‐SLBP(18‐126)‐IRES‐H1‐mMaroon1 construct, and the rest to be undetermined. Note that a negative signal can be caused by either cell cycle progression or low or no expression of the plasmid in the cells, so it will not decide on cell cycle stages purely based on negative signals. Finally, cell cycle stages were determined based on the following criteria: G1(0) –mKO2+, Clover‐, S–Clover+, mTurquoise2+ or mKO2+, G2–mTurquoise2‐, mKO2‐, mMaroon1+ but not condensed, M–mTurquoise2‐, mKO2‐, mMaroon1+ and condensed.

For each identified cell nucleus, the outline of the corresponding cell was then drawn manually using a custom ImageJ macro. The average cytoplasmic cyclin B1 intensity was then measured and compared against the cell cycle stages determined by the nuclear Fucci4 signals.

### Quantification of Cell Cycle Distribution Along Invading Strands

Spheroids and organoids were allowed to invade collagen for 2–3 days to form a sufficient number of invading strands and then labeled with proliferation markers as described above. For spheroids with mitochondrial function inhibited, 20 µm antimycin A was added to the growth medium 24 h before fixation. Individual invading strands containing clearly identifiable leader and follower cell nuclei were blindly selected based on DAPI staining and bright‐field images. For each selected strand, cells were assigned an order based on the position of the nuclear center along the strand (Figure 2c). As it becomes progressively more difficult to distinguish between different cell nuclei at rear positions than at frontal positions, only up to the first eight physically connected cells in each strand with clearly identifiable nuclear position were quantified, and the fifth to eighth cells were grouped together. For each nuclear marker (i.e., EdU, H3S10p, Ki‐67), a cell is blindly identified as positively stained if more than half of the nuclear area exhibits fluorescent signals after background subtraction. For the cytoplasmic marker (cyclin B1), a cell is considered positively stained if the region surrounding the nucleus exhibits fluorescent signals after background subtraction. Only cells that can be unambiguously identified as positively or negatively stained were included for analysis. A percentage of positively stained cells was then calculated for each position order or the fifth to eighth‐order group along the invading strand for each proliferation marker. EdU positive (EdU+) cells were considered to be in the S phase, H3S10p positive (H3S10p+) cells were considered to be in the M phase, Ki‐67 positive (Ki67+) cells were considered to be in an active cell cycle (i.e., non‐G0 phase), whereas Cyclin B1 positive and H3S10p negative (CB1+ H3S10p‐) cells were considered to be in the G2 phase of the cell cycle. A G2 phase cell was considered to be in the early G2 phase if it is also EdU+ suggesting that it enters the G2 phase during the EdU incubation period and was considered to be in the late G2 phase if it is EdU‐ suggesting that it enters the G2 phase after the EdU incubation period.

Alternatively, Fucci4‐expressing spheroids were allowed to invade collagen and form invading strands as described above before fixation. For spheroids with glycolysis inhibited, 25 mm 2‐DG was added to the growth medium 24 h before fixation. Individual invading strands containing clearly identifiable leader and follower cell nuclei were selected based on Fucci4 signals and bright‐field images. For each cell along the strand (up to the first eight cells), cell cycle stages were determined manually based on the criteria described above.

Note that unlike the 2D case described above, because of the scattering of light by objects on the optical path and the resulting attenuation of fluorescent intensity based on both the axial and lateral location of the cell within the 3D spheroids/organoids,^[^ ^8^ ^]^ an algorithm was not used to determine a threshold value to determine positive or negative labeling, but instead determined it manually by comparing with a neighboring cell that is unambiguously positive or negative as a reference. To be conservative, any cell that cannot be unambiguously determined was not included in the analysis.

### Quantification of Cell Cycle Distribution at the Wound Edge

MDA‐MB‐231 and 4T1 cells were seeded on glass substrates and allowed to grow until they were confluent. Several parallel wounds were created by scraping off a strip of cells with a 1000‐µL pipette tip as described previously.^[^ ^52^ ^]^ The sample was washed with PBS and replaced with complete growth medium and incubated for 6–24 h before fixation. 2 h before fixation, the cells were incubated with 10 µm EdU for 2 h at 37 °C. Following EdU incubation, cells were immediately fixed with 3.7% formaldehyde in PBS and permeabilized with 1% Triton X‐100. The cells were then labeled to visualize proliferation markers (EdU, cyclin B1, H3S10p) and the nuclei (DAPI) as described above.

Cells at the wound edge were then imaged for proliferation marker and DAPI signals. For each wound‐edge image, the image was first background‐subtracted and rotated to align the wound edge to be vertical. The aligned wound‐edge image was then projected in the direction of the edge to get the total intensity of each signal channel as a function of distance to the wound edge. To normalize the difference in cell density, each of the EdU, cyclin B1, and H3S10p channels was divided by the DAPI channel to get the normalized distribution of proliferation as a function of the distance to the wound edge.

### Quantification of Cell Division Frequency

Spheroids expressing the CycleTrak cell cycle indicator were used to quantify the division frequency along the strands. Cell division events were identified by examining the time‐lapse video recording (Figure 2f; Video S2, Supporting Information). The time of the frame containing a cell with a condensed chromosome immediately before its chromosome segregation was considered the time of cell division for the identified cell. The position of the nuclear center at the time of cell division and the position of the strand tip were recorded in ImageJ to determine the distance of cell division relative to the tip. To calculate cell division frequency, the strand was binned into several segments based on the relative distance to the tip of the strand. For each binned strand segment, the total number of cell division events that occurred within this segment was divided by the total time‐lapse duration of the segment, which was then divided by the bin length to normalize the bin size effect.

Note that the relative distance of cell division was used here instead of the relative order as used for the proliferation markers because the cell order is difficult to determine for cell divisions taking place far away from the tip of the strand in time‐lapse movies. To compare the two metrics, the distance of the nuclear center to the tip of the strand was measured for cells with identifiable relative order along the strand (Figure 2h). This relationship was then used to predict or estimate the relative order of the tracked cell.

### Artificial Mitochondria Transfer and Hybrid Spheroid Generation

External mitochondria were manually transferred to MDA‐MB‐231 cells as described previously.^[^ ^8^, ^53^ ^]^ Briefly, the mitochondria Isolation Kit for Culture Cells (Catalog # 89874, Thermo Scientific) was used to isolate mitochondria from donor CAFs following the manufacturer's instructions. The isolated mitochondria were resuspended in 50 µL of PBS and kept on ice before transfer. A DC protein assay (Catalog # 5000116, Bio‐Rad Laboratories) was used to determine the concentration of resuspended mitochondria. To increase membrane permeability, the recipient MDA‐MB‐231 cells were pre‐incubated with 20 mg mL^−1^ Pluronic F‐68 (Catalog # 24040032, Life Technologies) for 2 h before being harvested and resuspended in PBS. 0 or 1 µg donor mitochondria were then added to a centrifuge tube containing 100 000 recipient cells, and transferred to the cells by centrifugation at 1500 × g, 4 °C for 5 min. The recipient cells were then labeled with the CellTracker Green CMFDA (Catalog # C7025, Invitrogen) or CellTracker Orange CMRA dye (Catalog # C34551, Invitrogen) and mixed at a 1:1 ratio to generate hybrid spheroids.

### Tracking of Cell Migration in Strands

For the migration of cells in strands before cell division, the time and position of cell division along invading strands were determined as described above. For each dividing cell, the position of its nuclear center before the division was backtracked using a custom ImageJ macro until a frame that its nuclear position is no longer identifiable or until the first frame of the movie.

For migration of cells labeled with the Fucci4 probe for G2 versus G1(0)/S cells or cells labeled with different colors of the CellTracker dyes and Hoechst 33342 for cells with or without external mitochondria, the nuclear center of a clearly identifiable cell was tracked using ImageJ until its nuclear position is no longer identifiable or until the last frame of the movie.

The position of the tip of the strand was simultaneously tracked, and the relative localization of the nuclear center to the stand tip was then calculated. This relative distance was then used to quantify the migration of cells approaching the strand tip.

### Quantification of Spheroid Outgrowth

Spheroids were incubated in 10 µm CellTracker Orange CMRA dye for 20–30 min at 37 °C with 5% CO_2_, followed by twice medium wash before seeding into 4.5 mg mL^−1^ collagen for the outgrowth assay. Spheroid outgrowth was quantified by the spheroid migration index, which was calculated by measuring the projected spheroid area immediately after embedding in collagen (A_0_) and the projected spheroid area following culture in collagen (A_t_). Invasive Index was defined as (A_t_/A_0_ – 1).

### Statistical Analysis

Nonparametric statistical tests were used unless data were tested to be normal and variances were similar for the groups being compared. Two‐sided tests were used wherever applicable. Statistical significance was identified if the tested *p*‐value was smaller than 0.05(*), 0.01(**), 0.001(***), or 0.0001(****). When multiple pairwise comparisons were performed, an appropriate correction method was employed to adjust the significance level. All statistic tests were performed with Prism 8 (GraphPad Software, La Jolla, CA). Data are presented as mean ± S.E.M., Tukey's box plot (with the box representing the lower quartile, median, and upper quartile, the whiskers extending to the most extreme data points not considered outliers, and the individual data points representing outliers), or violin plot.

### Mathematical Modeling and Computer Simulation

Instead of modeling the migration and proliferation of individual cells, a RAD model was built to simulate and help understand the collective dynamics along quasi‐1D strands that involve the interplay of cell migration and proliferation. The geometry of the strand was modeled as a 1D ray along the positive *x*‐axis with the tip of the strand fixed at the origin (Figure 3a). Cells within the strand were assumed to move along this 1D ray, which was supported by experimental observations (Video [Link], [Link], [Link], [Link], Supporting Information). This *x*‐axis reference frame moved with the tip of the strand, such that no cells would move to a location with *x* < 0. For simplicity, the width of the strand and size of the cells were considered to be a constant and thus not included in the model. Since the effect of strand width and cell size was not considered in this simple model, the total cell density at each position along the *x*‐axis is a constant such that the density of each cell cycle phase was proportional to the fraction of the specific cell cycle phase, *ρ_i_*, where *i* = *G0*, *G1*, *S*, *G2*, or *M*, and the fractions sum to *1*.

At a given location *x* (*x* ≥ 0) and time *t*, the fraction or normalized density of each cell cycle phase *ρ_i_* (*x*,*t*) was controlled by the random migration of cells belonging to this cell cycle phase described by the diffusion coefficient *D_i_*, the directional migration of cells described by the directed migration or advection speed, *V* + *V_i_*, the new entry of cells from the previous cell cycle phase described by the exponential rate *e_i_*, and the exit of cells to the next cell cycle phase described by *e_i+1_*. As the dependence of random cell migration on the cell cycle phase is not the focus of this study, the diffusion coefficient was assumed a constant for all cell cycle phases, such that *D_i_* = *D*. The advection speed is separated into two components, the passive speed *V*, which is the same for all cell cycle phases, and an active component, *V_i_*, which accounted for cell cycle‐specific directional migration. The passive component is required for the continuous RAD model by the law of conservation. Newly generated cells from proliferation and other factors including directed migration toward the tip and apoptosis may increase or change the local cell density, such that all cells will be passively pushed away or toward the tip of the strand to maintain a constant local cell density. For proliferation, exponential rates of cell cycle progression for simplicity were mainly used, which assumed that the entry and exit of a specific cell cycle phase were equally distributed along the duration of the phase. Other more complicated proliferation models,^[^ ^32^ ^]^ such as the Smith–Martin type or age‐structured model, were also used, but complex proliferation models were not the focus here and were not likely to change the overall model results (Figure S5g, Supporting Information). The cells were modeled to sequentially progress through G0, G1, S, G2, and M for simplicity. However, the G0/G1 reversible transition might not be as straightforward.^[^ ^44^ ^]^ Cells might also modeled to sequentially progress through G1, S, G2, and M, and with an additional model to consider the reversible G0/G1 transition, but the model would be more complicated and not likely to qualitatively change the front‐rear cell cycle profiles. The control equations are shown below, assuming a cell sequentially progresses through G0, G1, S, G2, and M phases.

(5) $$\frac{\partial \rho_{i}}{\partial t}=\frac{\partial }{\partial x}\left(D\frac{\partial \rho_{i}}{\partial x}\right)-\frac{\partial \left(\left(V+V_{i}\right)\rho_{i}\right)}{\partial x}+a_{i}e_{i}\rho_{i-1}-b_{i}e_{i+1}\rho_{i}+c\rho_{i}$$

(6) $$\sum_{i}\rho_{i}=1$$

here, *a_i_* = 1, *b_i_* = 1, for all *i*, except that *a_G0_* = 2 to account for the generation of two daughter cells through cell division. An additional reaction source term described by *c* was also included in Equation (5) to account for possible contributions from additional states, for example, cell apoptosis and lateral expansion of the 1D geometry. The additional term was out of the scope of this study and was only included for validation purposes to demonstrate that the simulation result was robust against these additional variations when kept in a reasonable range (Figure S5, Supporting Information). Hence, *c* = 0 unless otherwise stated for simplicity.

The boundary conditions for the model assumed no net cell migration across the boundary at the tip (*x* = 0) for each cell cycle phase, and the distribution of normalized density to reach a stable value at a distance far away from the tip of the strand (*x = x_max_*) and are given by the equations below.

(7) $$\left\{\begin{array}{c} D\frac{\partial \rho_{i}}{\partial x}-\left(V+V_{i}\right)\rho_{i}=0,x=0\\ \frac{\partial \rho_{i}}{\partial x}=0,x=x_{\max}>>0 \end{array}\right.$$

Summing Equation (5) across all *i*, one obtains the following equation

(8) $$\frac{\partial \sum_{i}\rho_{i}}{\partial t}=\frac{\partial }{\partial x}\;\left(\;D\frac{\partial \sum_{i}\rho_{i}}{\partial x}\right)\;-\;\frac{\partial \left(V\sum_{i}\rho_{i}+\sum_{i}V_{i}\rho_{i}\right)}{\partial x}+e_{G0}\rho_{M}+c\sum_{i}\rho_{i}$$

Substituting Equation (8) with (6), resulted in

(9) $$\frac{\partial \left(V+\sum_{i}V_{i}\rho_{i}\right)}{\partial x}=e_{G0}\rho_{M}+c$$

Combining Equations (6), (7), and (9), resulted in

(10) $$V+\sum_{i}V_{i}\rho_{i}=\int_{0}^{x}\left(e_{G0}\rho_{M}+c\right)dx$$

or

(11) $$V\left(x\right)=\int_{0}^{x}\left(e_{G0}\rho_{M}+c\right)dx-\sum_{i}V_{i}\rho_{i}$$

To enable tracking of how much time the cells have spent in the G2 phase, an extra age‐structured variable,^[^ ^32^ ^]^ *τ* (*τ* ≥ 0), was added to the control equation for G2 cells, making it become Equation (4) in the main text, which is also listed below,

(12) $$\begin{array}{ccc} \frac{\partial \psi_{G2}}{\partial t} & + & \frac{\partial \psi_{G2}}{\partial \tau }=\frac{\partial }{\partial x}\left(D\frac{\partial \psi_{G2}}{\partial x}\right)-\frac{\partial \left(\left(V+V_{G2}\right)\psi_{G2}\right)}{\partial x}\\ & - & {e_{M}}^{\prime }\psi_{G2}\left(\tau >\tau^{\prime }\right)+c\psi_{G2} \end{array}$$

here, *τ* is the age of the G2 cells, i.e., the time a cell has spent in the G2 phase, and ψ_*G*2_(*x*,*t*, τ) is the fraction of G2 cells with an age of *τ* at time *t* and location *x*, τ′ is a refractory period within which the cell would not enter the next cell cycle phase,^[^ ^32^ ^]^ and *e_M_* ′ is the cell cycle transition rate for non‐refractory G2 cells. The integration of *ψ* over *τ* gives the total fraction of G2 cells, $\rho_{G2}\left(x,t\right)=\int_{\tau \geq 0}\psi_{G2}\left(x,t,\tau \right)d\tau$. G2 cells at any age τ > τ′ can enter the M phase with exponential rate *e_M_* ′, but only G2 cells at the age of *τ* = 0 are connected with cells entering the G2 phase from the S phase, such that ψ_*G*2_(*x*,*t*, 0) = *e*_*G*2_ρ_*S*_(*x*,*t*).

The control equation for M‐phase cells will also be rewritten as

(13) $$\begin{array}{c} \frac{\partial \rho_{M}}{\partial t}=\frac{\partial }{\partial x}\left(D\frac{\partial \rho_{M}}{\partial x}\right)-\frac{\partial \left(\left(V+V_{M}\right)\rho_{M}\right)}{\partial x}+{e_{M}}^{\prime }\rho_{G2\left(\tau >\tau^{\prime }\right)}-e_{G0}\rho_{M}+c\rho_{M}\;\; \end{array}$$

The parameters of the model are estimated below.

The cell cycle transition rate *e_i_* (*x*) was first assigned with a constant value determined from the experimentally observed fraction of each cell cycle phase at the right end (*x* = *x*_max _in the model and fifth to eighth positions in experiments), such that the model would eventually reach a steady state with these experimentally observed fractions, ρ_*i*_^*stable*^, if only proliferation (and other reaction source terms) and passive advection are simulated in the model. Hence, the result is

(14) $$\left\{\begin{array}{c} e_{G0}\left(x\right)=\frac{1}{{\rho_{M}}^{stable}}e_{Base}\\ e_{G1}\left(x\right)=\frac{2-{\rho_{G0}}^{stable}}{{\rho_{G0}}^{stable}}e_{Base}\\ e_{S}\left(x\right)=\frac{2-{\rho_{G0}}^{stable}-{\rho_{G1}}^{stable}}{{\rho_{G1}}^{stable}}e_{Base}\\ e_{G2}\left(x\right)=\frac{2-{\rho_{G0}}^{stable}-{\rho_{G1}}^{stable}-{\rho_{S}}^{stable}}{{\rho_{S}}^{stable}}e_{Base}\\ e_{M}\left(x\right)=\frac{2-{\rho_{G0}}^{stable}-{\rho_{G1}}^{stable}-{\rho_{S}}^{stable}-{\rho_{G2}}^{stable}}{{\rho_{G2}}^{stable}}e_{Base} \end{array}\right.$$

here, *e_Base_* is a constant base rate representing the new cells generated from cell division and other factors including cell death at each unit time, and it could be estimated from the average doubling time of cells undergoing exponential growth, *t_double_*, such that *e_Base_* = ln 2/*t_double_*.

The age‐structured G2 density will decrease with a rate of *e_Base_* due to passive advection to meet Equation (6) within the refractory period τ′ and with a rate of *e_Base_* + *e_M_* ′ thereafter. Hence, at steady state, it will approximately have the following age‐structured density,

(15) $$\begin{array}{c} \psi_{G2}\left(\tau \right)=\left\{\begin{array}{c} \psi_{G2}\left(0\right)\exp\left(-e_{Base}\tau \right),0<\tau <\tau^{\prime }\\ \psi_{G2}\left(0\right)\exp\left(-e_{Base}\tau^{\prime }\right)\exp\left(-\left(e_{Base}+{e_{M}}^{\prime }\right)\left(\tau -\tau^{\prime }\right)\right),\tau \geq \tau^{\prime } \end{array}\right. \end{array}$$

To maintain the same stable state condition, it is required that the fraction of cells entering the next phase be independent of the refractory period, such that *e_M_* ρ_*G*2_^*stable*^ = *e_M_* ′ρ_*G*2(τ > τ′)_^*stable*^. Hence, it resulted in

(16) $${e_{M}}^{\prime }=\frac{e_{M}e_{Base}}{\left(e_{Base}+e_{M}\right)\exp\left(-e_{Base}\tau^{\prime }\right)-e_{M}}$$

To account for the experimental observation that cell proliferation decreases in the proximity of the strand tip, an error function was assigned to the G0‐to‐G1 phase transition rate or the cell cycle entry rate given by Equation (2) in the main text. In Equation (2), *erf*(*x*) is an error function with respect to *x*, whereas *c_1_* and *c_2_* are constants determined empirically such that S cells and Non‐G0 cells from simulation have quantitatively similar distributions as that from experiments, and $c_{3}=\frac{2-{\rho_{G0}}^{stable}}{{\rho_{G0}}^{stable}}e_{Base}$ is the stable value of *e*_*G*1_(*x*) at *x* = *x*_max _ ≫ 0 as determined from Equation (14). *D* is derived from the measurement of random migration speed (Figure S6f, Supporting Information) in spheroids or organoids, and approximated by the formula, *D* = *rmsd* ^2^/2Δ*t*, where *rmsd* is the root‐mean‐square displacement measured with time interval Δ*t*.

The parameters used for the simulations are listed in Table S1 (Supporting Information). Computer simulation of the above model was carried out using MATLAB. To start the simulation, an arbitrary initial condition was given such that *ρ_i_* (*x*,0) satisfies Equation (6).

### Code Availability

All codes used in this study, including custom scripts for data analysis and computer simulation, are described in the Experimental Section and are available from the corresponding authors upon reasonable request.

## Conflict of Interest

The authors declare no conflict of interest.

## Author Contributions

J.Z. designed experiments, performed experiments, analyzed data, developed the computational model, and wrote the manuscript; J.A.M., Y.W., L.W., P.V.T., W.W., and H.S. performed experiments; C.A.R.‐K. supervised the study and edited the manuscript.

## Supporting information

Supporting Information

Supplemental Video 1

Supplemental Video 2

Supplemental Video 3

Supplemental Video 4

Supplemental Video 5

## Data Availability

The data that support the findings of this study are available from the corresponding author upon reasonable request.
