# Supplementary material for: Cellular Energy Cycle Mediates an Advection‐Like Forward Cell Flow to Support Collective Invasion
Source: Adv Sci (Weinh). 2024 Jun 21;11(32):2400719. doi: 10.1002/advs.202400719 (PMC11348062; doi:10.1002/advs.202400719)
Supplement: Supplementary file 1 — Supporting Information [file ADVS-11-2400719-s005.docx]

Supporting Information

Cellular Energy Cycle Mediates an Advection-Like Forward Cell Flow to Support Collective Invasion

*Jian Zhang*, Jenna A. Mosier, Yusheng Wu, Logan Waddle, Paul V. Taufalele, Wenjun Wang, Heng Sun, and Cynthia A. Reinhart-King**

Supplementary Figures


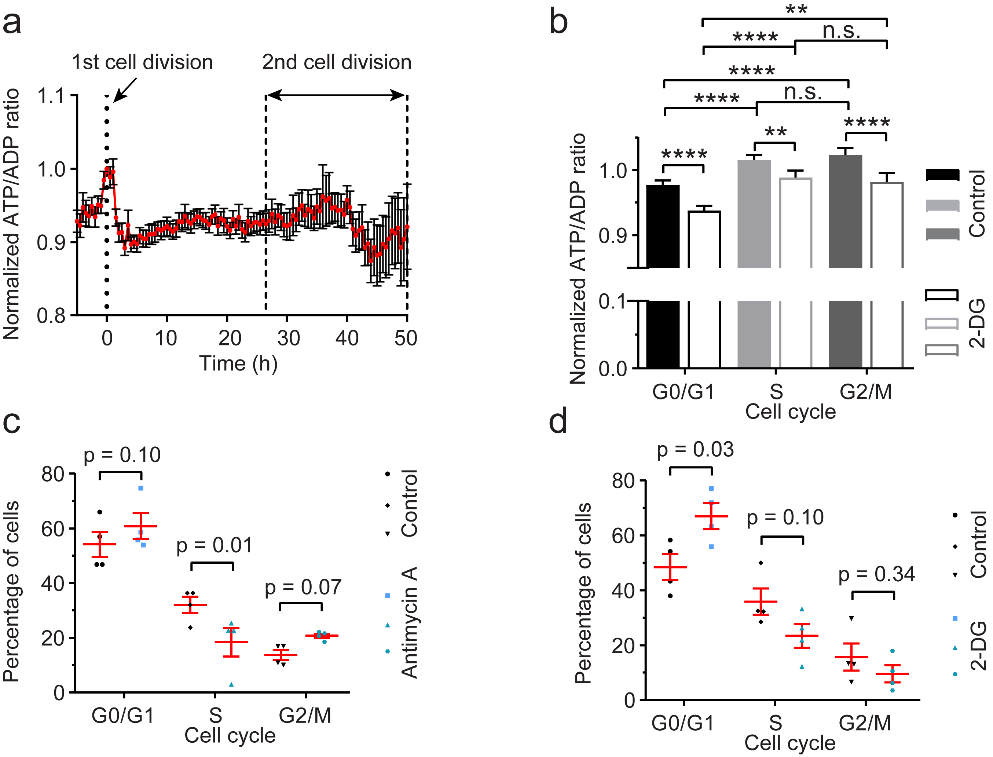


**Figure S1. Cell cycle regulated bioenergetics with metabolic inhibitors. (a)** Time-lapse data shows that cellular energy level drops following cell division and then continuously increases until the next cell division (N = 18 cells; data normalized to the mean at time = 0) in MDA-MB-231 cells. Vertical dotted line indicates time of first cell division, whereas vertical dashed lines indicate the range of second cell division. Related to Figure 1e. **(b)** Inhibition of glycolytic ATP production by 2-DG decreases ATP/ADP ratio as compared to control but does not eliminate the difference in ATP/ADP ratio between cells from different cell cycle phases (N = 6,114, 7,140, 5,169, 2,792, 2,830, and 2,045 cells for each group, respectively; data normalized to the mean of control). Both antimycin A **(c)** and 2-DG **(d)** have inhibitory effects on cell cycle progression. Data are pooled from three independent experiments. Error bar represents S.E.M. Scale bar, 25 µm. Statistical significance is tested by Kruskal-Wallis multiple comparison test with Dunn’s correction. ** p < 0.01, **** p < 0.0001, n.s. - not significant.


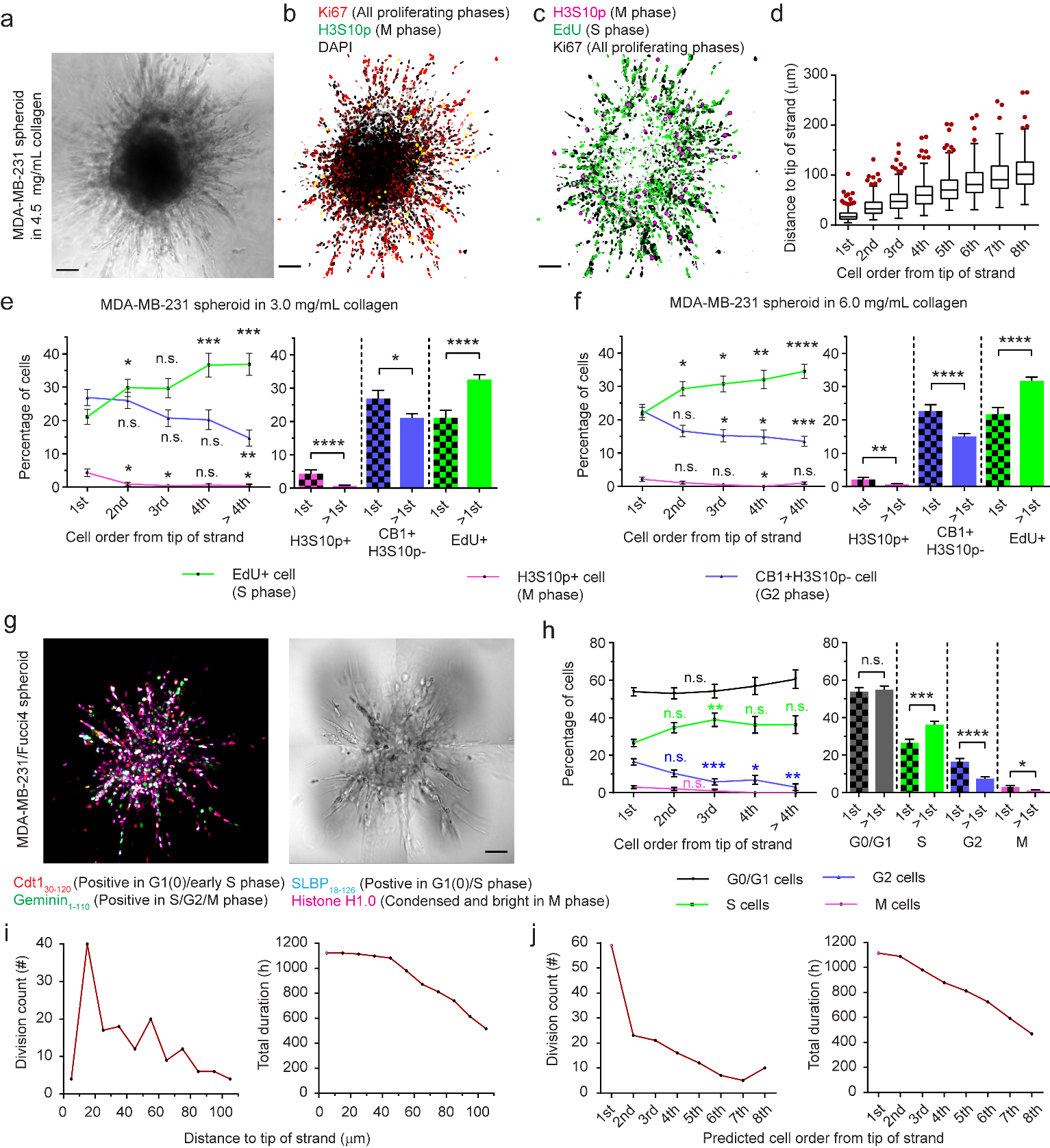


**Figure S2. The distribution of cell proliferation in tumor spheroids.** **(a)** Representative single-Z-stack bright-field image of an MDA-MB-231 tumor spheroid invading in 4.5 mg mL^-1^ collagen. **(b-c)** The spheroid is labeled with respective markers for proliferation and shown as maximum-intensity projected images across all confocal Z stacks. The same spheroid is also shown in Figure 2. **(d)** Distance to tip of invading strand as a function of cell order along the strand in spheroids. **(e-f)** Distribution of S (EdU+), G2 (CB1+H3S10p-), and M (H3S10p+) phase cells along invading MDA-MB-231 strands in 3.0 mg mL^-1^ (N = 327, 319, 256, 183, and 217 cells from 12 spheroids, respectively) and 6.0 mg mL^-1^ collagen (N = 472, 457, 387, 296, and 510 cells from 10 spheroids, respectively). **(g)** A representative max intensity projection image of an MDA-MB-231 spheroid expressing the Fucci4 probe invading in 4.5 mg mL^-1^ collagen on the left, with a corresponding single Z-stack bright-field image on the right. **(h)** Distribution of G0/G1, S, G2, and M phase cells determined by the Fucci4 probe along invading MDA-MB-231 strands in 4.5 mg mL^-1^ collagen (N = 501, 300, 190, 116, and 99 cells from 17 spheroids, respectively). **(i)** Cell division frequency is calculated based on the total cell division number observed within each 10-µm segment along the strands in spheroids and the corresponding total duration of observation of each segment. **(j)** The total division count and total duration as a function of cell order along the invading strands in spheroids. The cell orders are predicted from the distance to tip of the strand. Scale bar, 100 µm. Data are pooled from at least two independent experiments. Error bar represents S.E.M. Statistical significance is tested by Chi-square test for independence, followed by multiple comparisons to the leader (1st) group. Significances for multiple pair-wise comparisons are adjusted with the Bonferroni correction. * p < 0.05, ** p < 0.01, *** p < 0.001, **** p < 0.0001, n.s. - not significant.


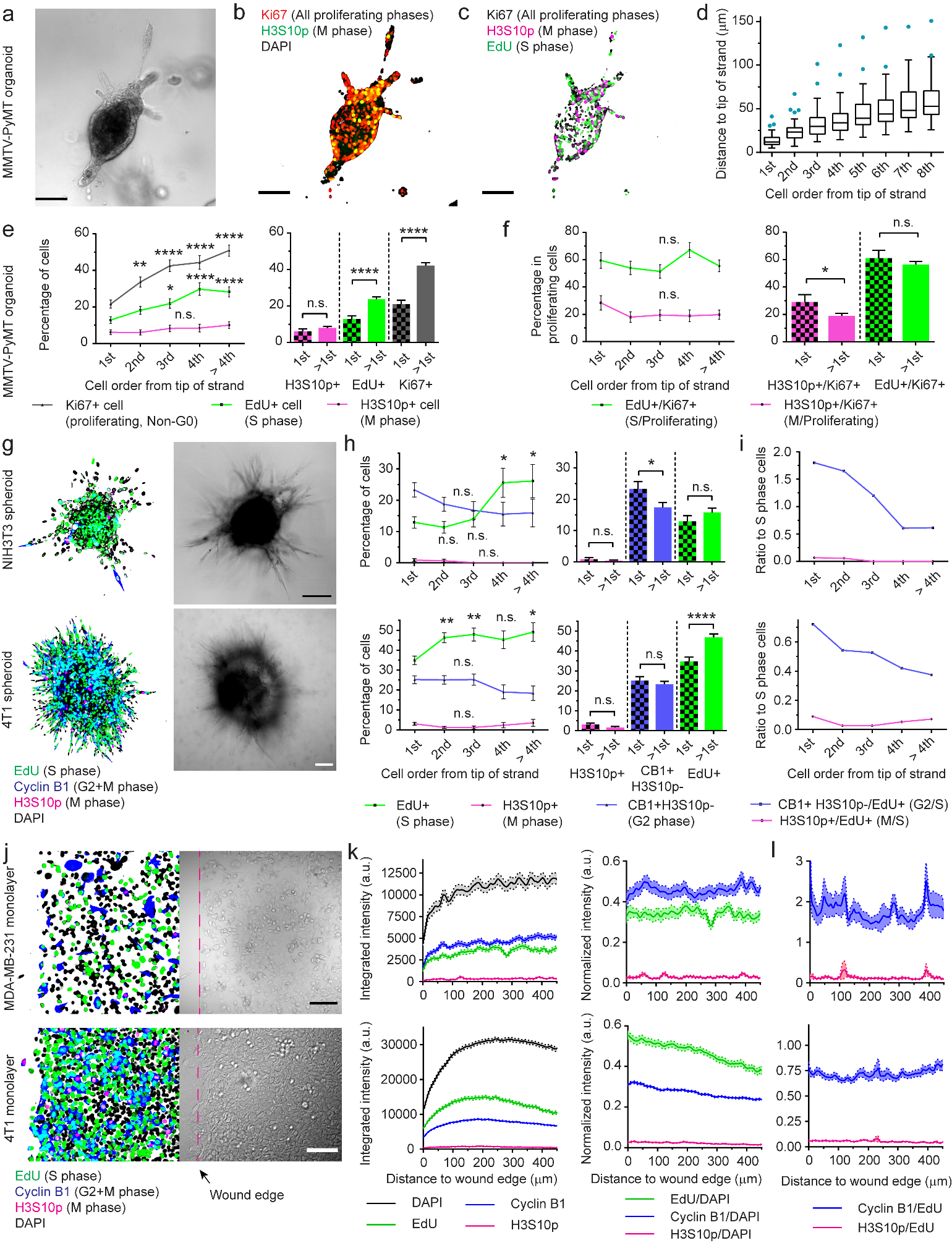


**Figure S3. Differential distribution of cell proliferation profile is a general property for 3D collective migration.** **(a)** Representative single-Z-stack bright-field image of an MMTV-PyMT tumor organoid invading in 4.5 mg mL^-1^ collagen. **(b-c)** The organoid is labeled with respective markers for proliferation and shown as maximum-intensity projected images across all confocal Z stacks. The same organoid is also shown in Figure 2. **(d)** Distance to tip of invading strand as a function of cell order along the strand in organoids. **(e)** S phase (EdU+) cells and proliferating cells (Ki67+) are significantly enriched in follower positions. N = 342, 297, 243, 180, and 279 cells from 69 organoids, respectively. **(f)** The relative percentage of S cells compared to all proliferating cells is independent of the cell order along the strands, whereas the relative percentage of M cells is significantly enriched in the leader position. N = 72, 97, 101, 80, 143 Ki67+ cells from 69 organoids, respectively. **(g)** Representative max intensity projection image of an NIH3T3 spheroid (top) and a 4T1 spheroid (bottom) invading in 3.0 mg mL^-1^ collagen labeled with respective proliferation markers, along with corresponding single Z-stack bright-field images of the spheroids on the right. **(h)** Distribution of S (EdU+), G2 (CB1+H3S10p-), and M (H3S10p+) phase cells along invading NIH3T3 (top) and 4T1 (bottom) strands in 3.0 mg mL^-1^ collagen (N = 348, 299, 179, 90, and 69 cells from 20 NIH3T1 spheroids, and N = 484, 398, 234, 126, and 114 cells from 25 4T1 spheroids, respectively). **(i)** In both NIH3T3 (top) and 4T1 (bottom) spheroids, G2 and M cells are more enriched at the leader position than S cells when calculating the ratios. **(j)** Representative images of MDA-MB-231 cells 6 h post wounding (top) and 4T1 cells 24 h post wounding (bottom) with respective labeling of proliferation markers, along with the corresponding bright-field images on the right. **(k)** Distribution of DAPI, CyclinB1, EdU, and H3S10p signals, as well as the signals normalized to the DAPI or EdU signal, as a function of the distance to the wound edge (N = 49 MDA-MB-231 images, and N = 144 4T1 images, respectively). **(l)** EdU, Cyclin B1, and H3S10p signals do not exhibit apparent differential enrichments concerning the distance to the wound edge when calculating the ratios. Scale bar, 100 µm. Data are pooled from at least two independent experiments. Error bar represents S.E.M. Statistical significance is tested by Chi-square test for independence, followed by multiple comparisons to the leader (1st) group. Significances for multiple pair-wise comparisons are adjusted with the Bonferroni correction. * p < 0.05, ** p < 0.01, *** p < 0.001, **** p < 0.0001, n.s. - not significant.


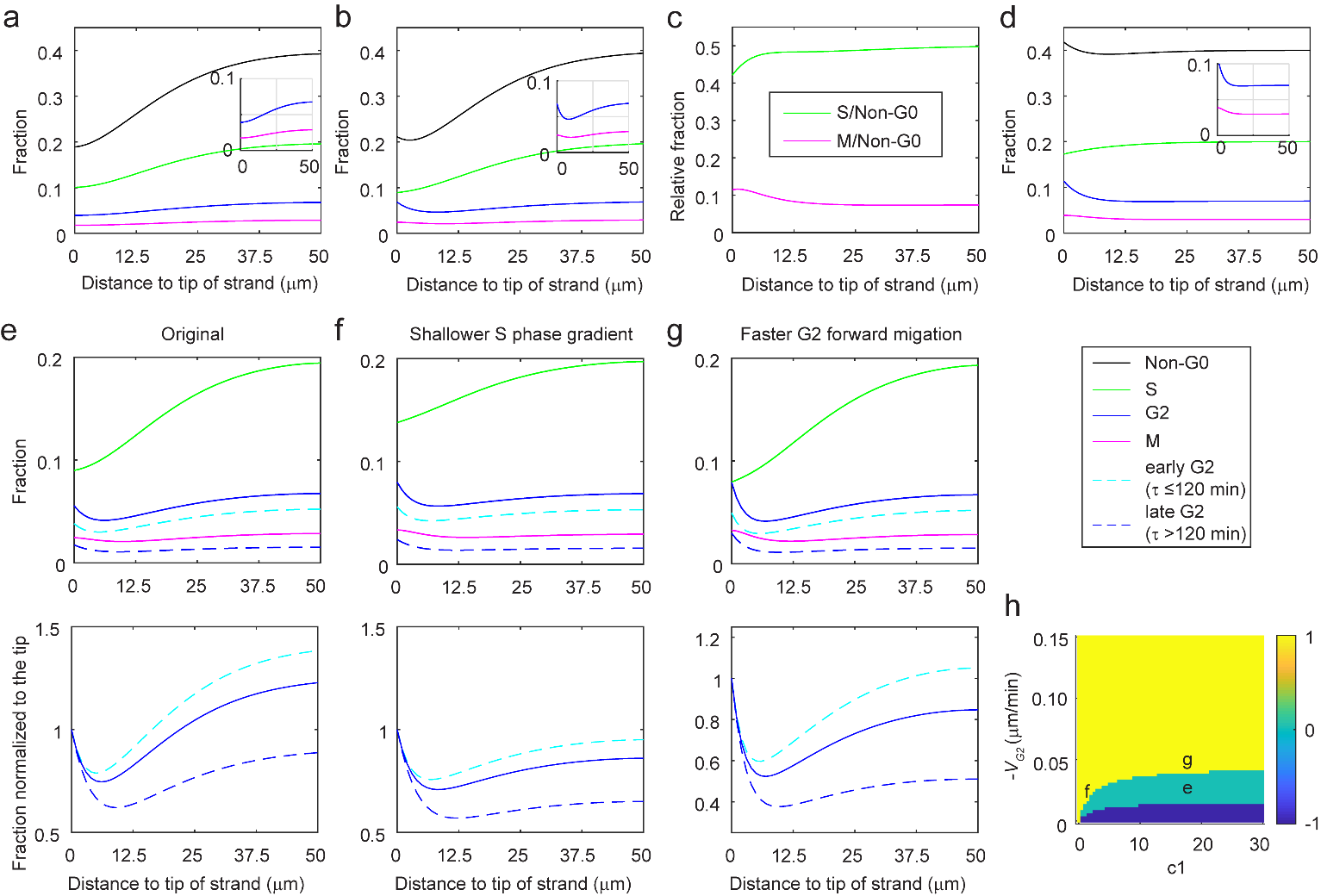


**Figure S4. Computer simulation of cell cycle distribution along invading strands in organoids. (a)** RAD model simulated distributions of proliferating (Non-G0), S, G2, and M cells in organoids with only location-dependent cell cycle entry. Model simulated distribution of proliferating (Non-G0), S, G2, and M cells **(b)** and relative distribution of S and M cells against all proliferating cells **(c)** in organoids with both location-dependent cell cycle entry and G2 cell forward motion. **(d)** Model simulated cell cycle distribution in organoids with only G2 forward motion. **(e-g)** Impact of model parameters on G2 cell distribution. A shallower S phase gradient **(f)** or a faster G2 cell forward migration speed **(g)** makes it easier for both early and late G2 cells to accumulate at the tip of the strand, as compared to the original model **(e)**. **(h)** Phase diagram shows the impact of model parameters on G2 cell distribution with marked points corresponding to parameters used in e-g (1 - both early and late G2 cells are front-concentrated, 0 - early G2 cells are rear-concentrated while late G2 cells are front-concentrated, -1 - both early and late G2 cells are rear-concentrated; here, a distribution is considered to be rear-concentrated if the fraction for more than half of the rear positions are larger than the tip position). The insets in **(a), (b),** and **(d)** highlight the distribution of G2 and M cells.


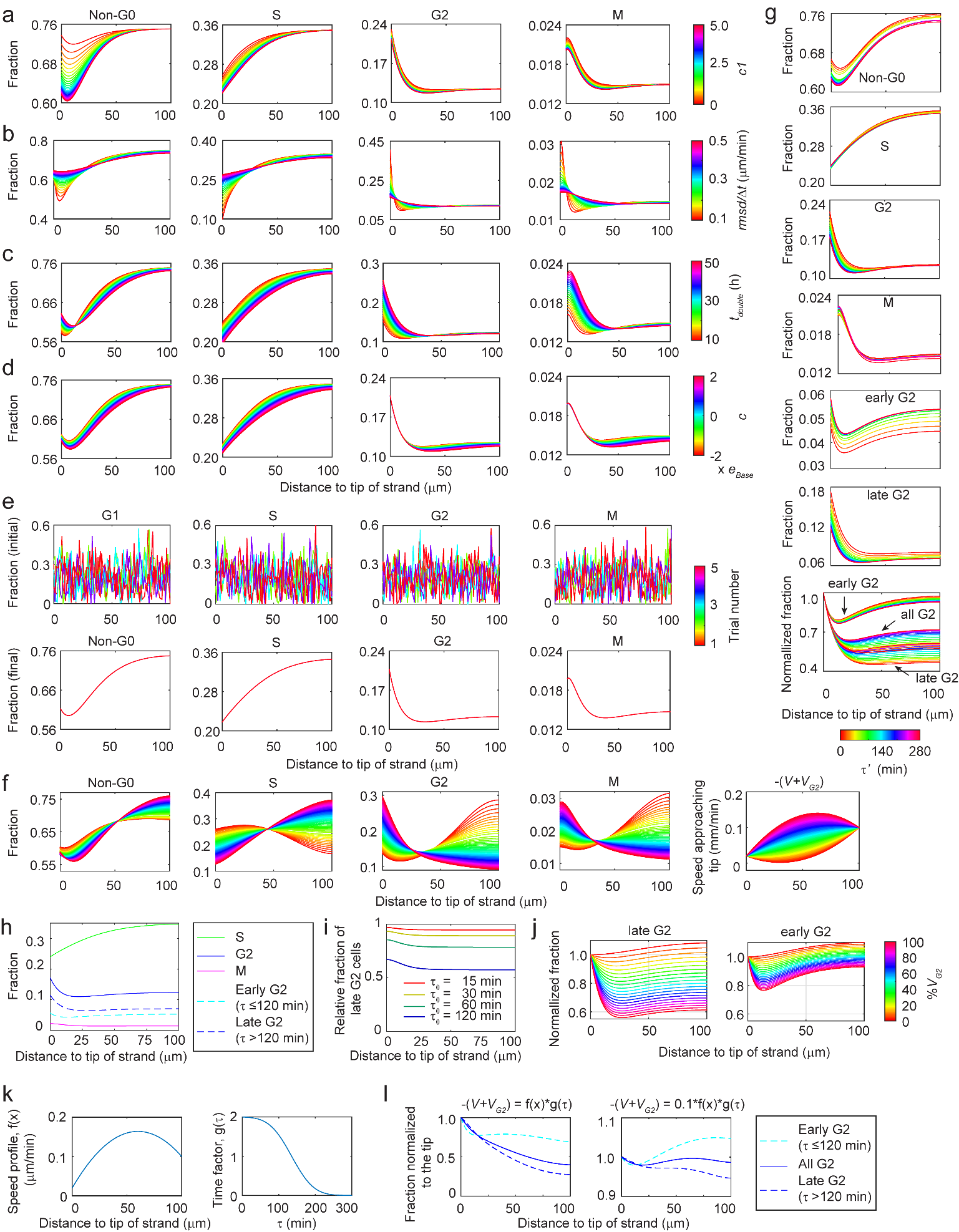


**Figure S5. Computer simulation of cell cycle distribution along invading strands in MDA-MB-231 spheroids.** The dependence of simulated cell cycle distribution on model parameters, including the location-dependent cell cycle entry described by the variable *c1* **(a)**, the random migration/diffusion speed described by the ratio of *rmsd/Δt* **(b)**, the cell doubling time *t_double_* **(c)**, additional reaction/source terms such as apoptosis described by the variable *c* **(d)**, arbitrarily assigned initial distributions **(e)**, the profile of the speed at which G2 cells approaching the tip described by the variable –(*V+V_G2_*) **(f)**, the refractory period of G2 cells described by the variable *τ’* **(g)**, and the percentage of reduced G2 speed described by the variable % *V_G2_* **(j). (h)** Model simulated distribution of S, G2, M cells, and early and late G2 cells. **(i)** The normalized fraction of simulated late G2 cells decreases with increasing *τ_0_* (EdU incubation period) in spheroids. **(k)** Speed profile as a function of distance to strand tip (*f(x)*) and the age of G2 cells (g(*τ)*) used to mimic the experimental observation in spheroids more closely. **(l)** Normalized distribution of early, late, and all G2 cells from model simulation using G2 speed profiles resemble that of experimental observation in control (left) and antimycin A treated condition (right).


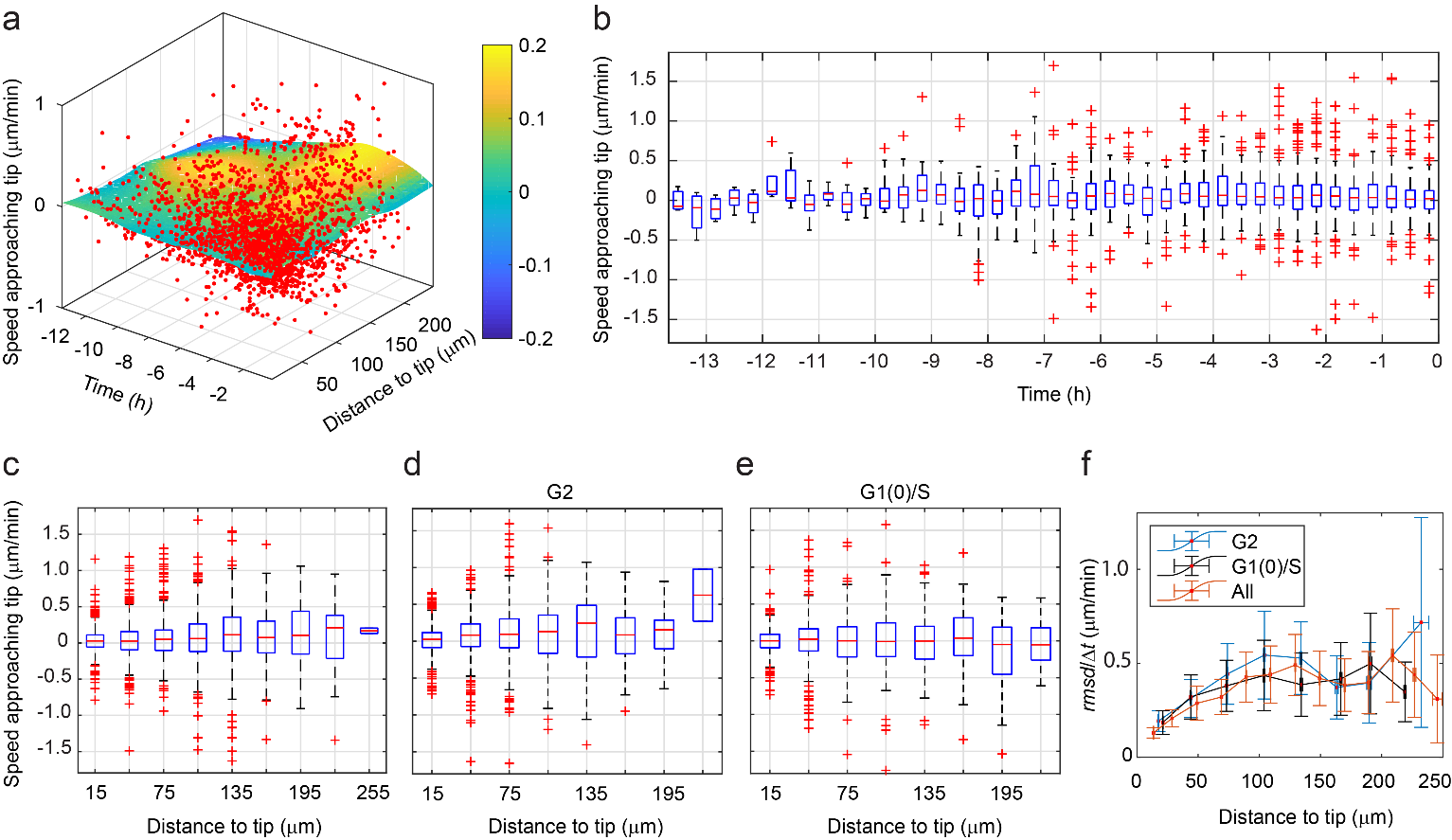


**Figure S6. Cells migrate toward the tip of the strand before division.** **(a)** Spatiotemporal distribution of cell migration speed approaching the tip of strand with regard to the distance to the tip and the time before cell division (t = 0). Each red dot indicates a measured data point (N = 2,645 data points), and the heatmap indicates a lowess fit of the measured data. **(b)** Distribution of migration speed approaching the tip at each time point before cell division (t = 0). **(c)** Distribution of migration speed approaching the tip before cell division within each 30-µm segment along the invasion strands. Distribution of migration speed approaching the tip for G2 cells **(d)** and G1(0)/S cells **(e)** within each 30-µm segment along the strands. **(f)** Root-mean square velocity as a function of distance to strand tip (N = 1,577, and 1,425, and 10,001 speed data points for G2, G1(0)/S, and all cells, respectively). Data are pooled from at least three independent experiments. Error bar represents S.E.M.


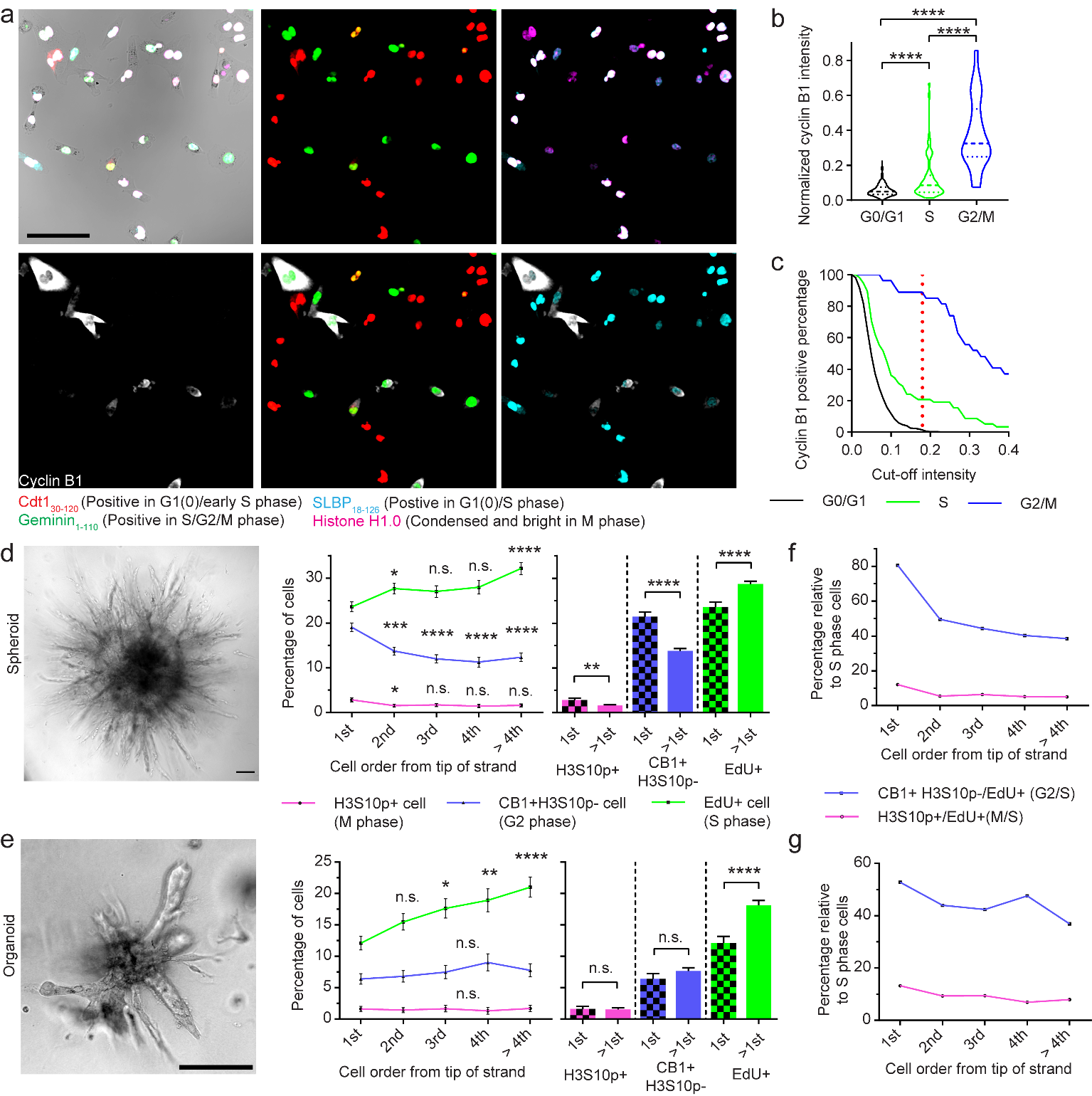


**Figure S7. Distribution of G2 cells in organoids and spheroids.** **(a)** Representative images of MDA-MB-231 cells expressing the Fucci4 probe on glass (top row: bright-field image overlayed with all four Fucci4 channels, two of the Fucci4 channels for G1/S transition, two of the Fucci4 channels for S/G2 transition) along with fluorescent signal of Cyclin B1 in the cells (bottom row: cyclin B1 alone, cyclinB1 overlayed with two of the Fucci4 channels, cyclinB1 overlayed with one of the Fucci4 channels). **(b)** Normalized fluorescent intensity of cyclin B1 in cells at various stages of the cell cycle determined by the Fucci4 probe (N = 342, 58, and 27 cells, pooled from three independent experiments, respectively). **(c)** The percentage of cells considered as positive for cyclin B1 signal as a function of the cut-off (threshold) intensity, with the red dotted line indicating a potential optimal threshold where most of the positive cells being G2/M cells, a small percentage being S cells, and little or no being G1(0) cells. G2 cells (CB1+ H3S10p-) are significantly enriched in the leader position in spheroids **(d)** but not organoids **(e)**. Single-Z-stack bright field images of the spheroid and organoid corresponding to the ones shown in Figure 5a and 5c are shown on the left side. N = 1,580, 1,514, 1,228, 897, and 1,247 cells for each cell order group from 22 spheroids, and N = 876, 762, 601, 454, and 656 cells for each cell order group from 174 organoids, pooled from at least two independent experiments, respectively. **(f-g)** In both spheroids and organoids, G2 and M cells are more enriched at the leader position than S cells. Scale bar, 100 µm. Statistical significance is tested by Kruskal-Wallis multiple comparison test with Dunn’s correction (**b**) or Chi-square test for independence followed by multiple comparisons to the leader (1st) group (**d**, **e**). Significances for multiple pair-wise comparisons are adjusted with the Bonferroni correction. Error bar represents S.E.M. * p < 0.05, ** p < 0.01, *** p < 0.001, **** p < 0.0001, n.s. - not significant.


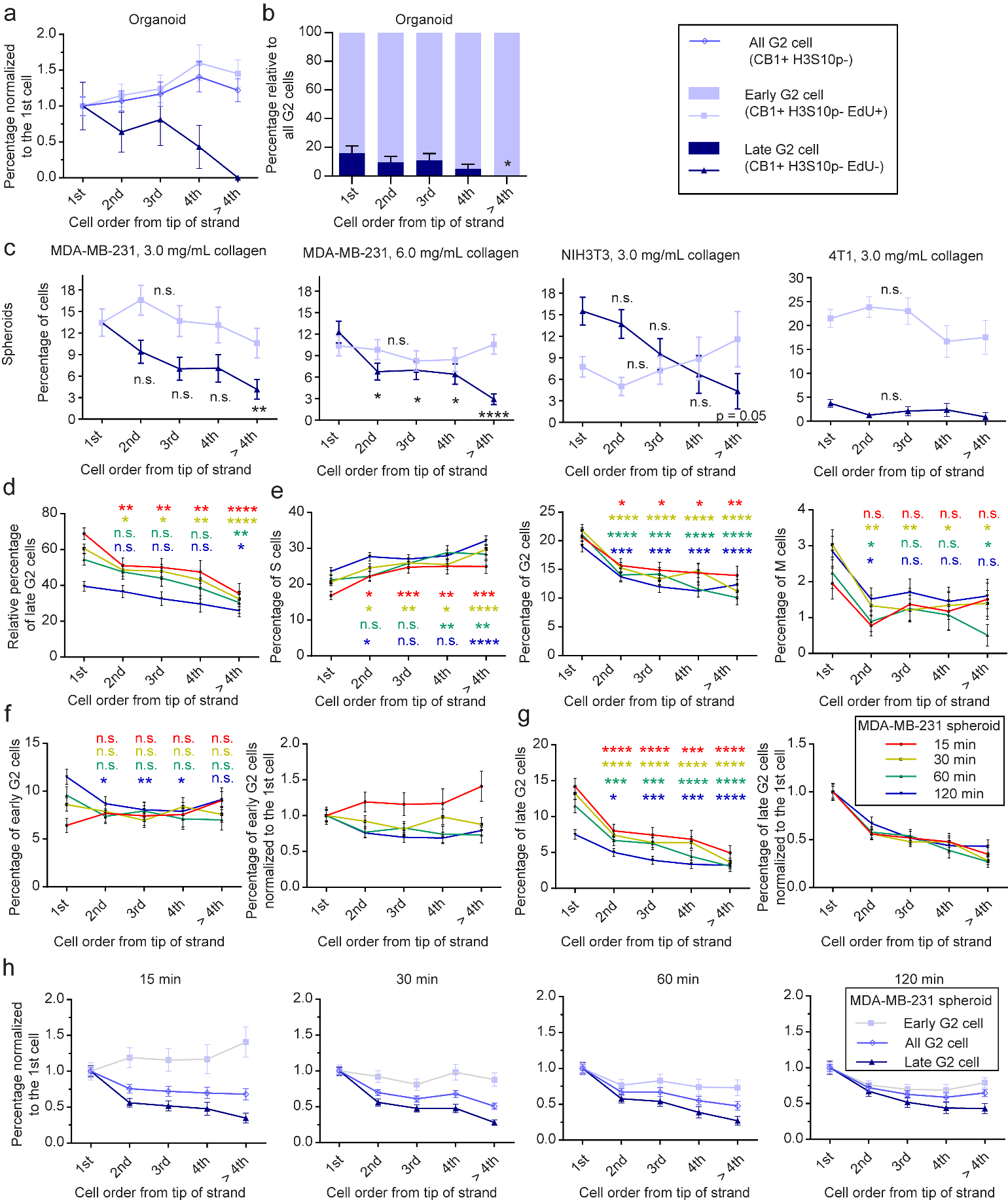


**Figure S8. Distribution of early and late G2 cells in organoids and spheroids.** **(a)** G2 distribution normalized to leader position in organoids shows that early and late G2 cells exhibit opposite trends of enrichment. **(b-d)** Late G2 cells are more enriched at the leader position than early G2 cells in organoids and spheroids. N = 876(56), 762(52), 601(45), 454(41), and 656(51) cells (G2 cells) for each cell order group from 174 organoids, N = 327, 319, 256, 183, and 217 cells from 12 MDA-MB-231 spheroids in 3.0 mg mL^-1^ collagen, N = 472, 457, 387, 296, and 510 cells from 10 MDA-MB-231 spheroids in 6.0 mg mL^-1^ collagen, N = 348, 299, 179, 90, and 69 cells from 20 NIH3T3 spheroids, and N = 484, 398, 234, 126, and 114 cells from 25 4T1 spheroids, respectively. **(e)** The distributions of S, G2, and M cells are independent of EdU incubation duration in spheroids. **(f-h)** Late G2 cells are always enriched at the leader position regardless of EdU incubation duration in spheroids, whereas early G2 cells switched from a rear-concentrated distribution to a front-concentrated distribution with the increase of EdU incubation duration. N = 1,027(212), 899(141), 658(98), 424(61), and 465(65); 1,650(360), 1,574(241), 1,236(165), 893(132), and 1,068(120); 1,157(244), 1,123(158), 882(125), 562(65), and 584(59); 1,580(301), 1,514(208), 1,228(147), 897(101), and 1,247(154) cells (G2 cells) for each cell order group from 20, 38, 28, and 22 spheroids for the 15 min, 30 min, 60 min, 120 min EdU incubation conditions, respectively. Data are pooled from at least two independent experiments. Statistical significance is tested by Chi-square test for independence, followed by multiple comparisons to the leader (1st) group. Significances for multiple pair-wise comparisons are adjusted with the Bonferroni correction. Error bar represents S.E.M. * p < 0.05, ** p < 0.01, *** p < 0.001, **** p < 0.0001, n.s. - not significant.


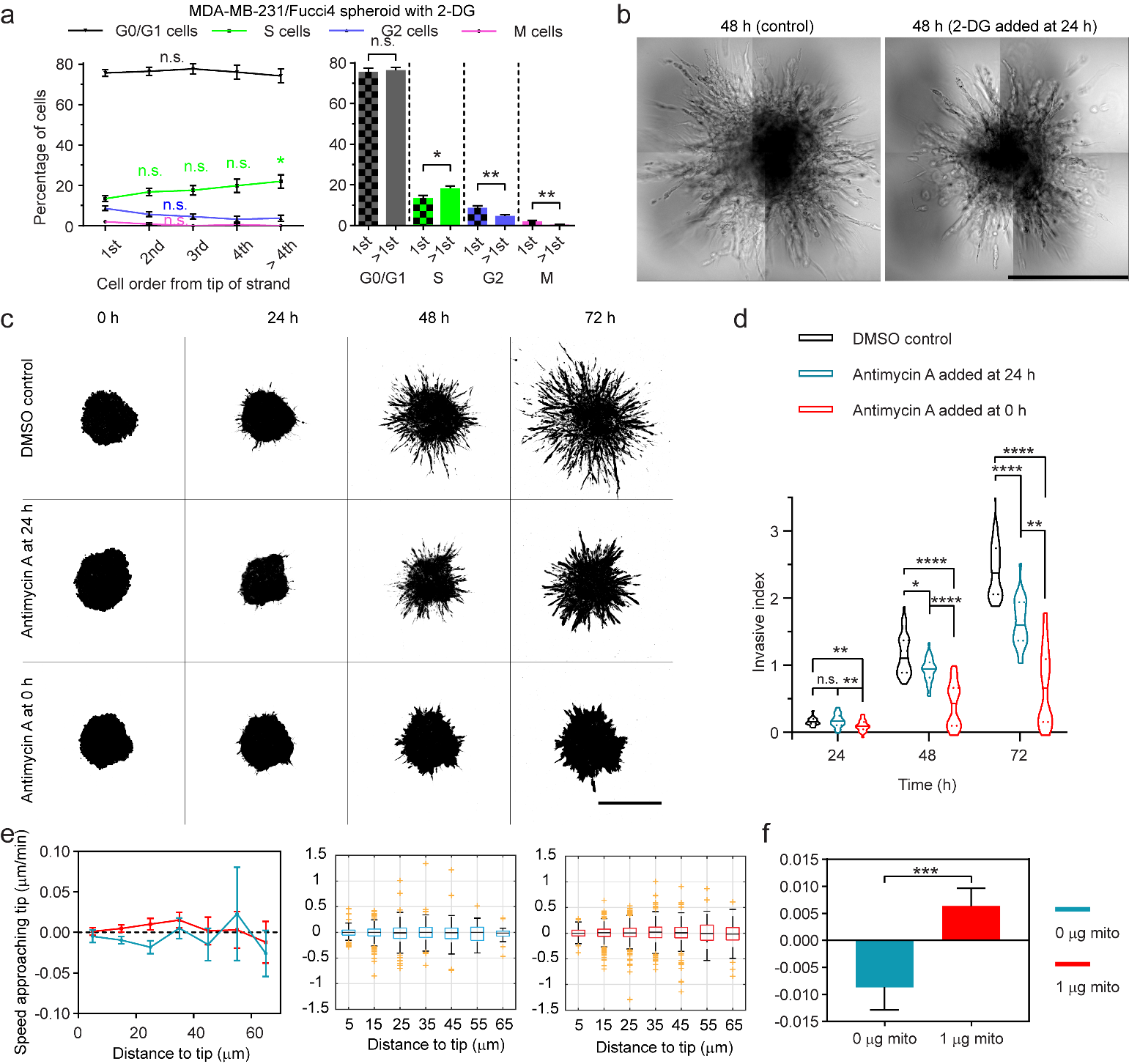


**Figure S9. Mitochondrial but not glycolytic ATP production contributes to G2 cell forward motion**. **(a)** Distributions of G0/G1, S, G2, and M phase cells determined by the Fucci4 probe along invading MDA-MB-231 strands in 4.5 mg mL^-1^ collagen treated with 25 mM 2-DG for 24 h (N = 621, 434, 261, 151, and 159 cells from 24 spheroids, pooled from at least two independent experiments, respectively). **(b)** 2-DG treatment inhibits spheroid invasion, as shown in the representative single-Z stack bright-field images of MDA-MB-231 spheroid invading in 4.5 mg mL^-1^ collagen. **(c)** Representative max intensity projection images of CellTracker-labeled MDA-MB-231 spheroids in 4.5 mg mL^-1^ collagen tracked at different time points post embedding. Spheroids are treated with antimycin A either immediately or 24 h post embedding. **(d)** Antimycin A treatment reduces the invasive migration of spheroids into surrounding matrices (N = 40, 33, 30, 40, 33, 26, 37, 30, and 24 spheroids for each group pooled from three independent experiments, respectively). **(e)** Cell migration speed approaching the tip as a function of distance to the tip in strands with both control cells and cells with external mitochondria (N = 103, and 122 tracked cells, respectively). **(f)** Cells with external mitochondria (1 µg mito) approach the tip with a positive average speed whereas control cells (0 µg mito) in the same strands migrate away from the tip on average (N = 1,751, and 2,541 speed data points, respectively). Scale bar, 500 µm. Statistical significance is tested by Chi-square test for independence followed by multiple comparisons to the leader (1st) group (**a**) with the Bonferroni correction, Kruskal-Wallis multiple comparison test with Dunn’s correction (**d**), or Mann-Whitney test (**f**). Error bar represents S.E.M. * p < 0.05, ** p < 0.01, *** p < 0.001, **** p < 0.0001, n.s. - not significant.

Supplementary Video Legends

**Video S1. Cell cycle mediated energetic dynamics.** A representative video of MDA-MB-231 cells showing the dynamics of normalized ATP/ADP ratio between cell divisions.

**Video S2. Cell division along collectively invading strands.** A representative video of an MDA-MB-231 spheroid expressing the CycleTrak cell cycle indicator (white indicates G0/G1 cells, cyan indicates S/G2/M cells) overlayed with the corresponding bright-field channel shows the typical location of cell divisions along the strands. Some of the identifiable divisions in the video are highlighted by red circles.

**Video S3. Cells move toward the strand tip before division.** A representative video of an MDA-MB-231 spheroid expressing the CycleTrak cell cycle indicator (white indicates G1/G0 cells, cyan indicates S/G2/M cells) overlayed with the corresponding bright-field channel shows the motion of the cells before division. Some of the identifiable divisions in the video are highlighted by red circles.

**Video S4. Migration of G2 vs G1(0)/S cells along an invading strand.** A representative video of an MDA-MB-231 spheroid expressing the Fucci4 cell cycle indicator (white indicates G1(0)/S cells, magenta indicates G2/M cells with M cells exhibiting a condensed morphology of the chromosome) overlayed with the corresponding bright-field channel shows the motion of the cells along an invading strand.

**Video S5. Migration of cells in a hybrid spheroid.** A representative video of an MDA-MB-231 spheroid composed of CellTracker Orange CMRA-labelled control cells (magenta) and CellTracker Green CMFDA-labelled cells with external mitochondria (green), with cell nuclei labeled with Hoechst 33342 in blue.

Supplementary Tables

Table S1. Parameters used in model simulations.

| Variable | Spheroid^[[1]](#footnote-2)^a) | Organoid |
| --- | --- | --- |
| *dt* [min] | 1 | 1 |
| $\tau'$[min]  | 270 | 90 |
| $t_{double}$[h]  | 29^[[2]](#footnote-3)^b) | 26^[[3]](#footnote-4)^c) |
| *dx* [µm] | 1 | 1 |
| $x_{\max}$[µm]  | 500 | 500 |
| $c$ | 0 | 0 |
| $c_{1}$ | 1 or 2 | 19 (1) |
| $c_{2}$ | 50 | 25 |
| $\rho_{G0}^{stable}$ | 0.25 | 0.60 |
| $\rho_{G1}^{stable}$ | 0.26 | 0.10 |
| $\rho_{S}^{stable}$ | 0.35 | 0.20 |
| $\rho_{G2}^{stable}$ | 0.125 | 0.07 |
| $\rho_{M}^{stable}$ | 0.015 | 0.03 |
| $V_{i},i=G0,G1,S,M$ | 0 | 0 |
| $V_{G2}$[µm min^-1^]  | -0.05 | -0.025^[[4]](#footnote-5)^d) (-0.05) |
| $\%V_{G2}$ | 20% (10%) | - |
| $rmsd/\Delta t$^e)^ [µm min^-1^]  | 0.27 | 0.135^d)^ |
| $\Delta t$[min]  | 20 | 20 |

References

[1] S. Seitz, R. Frege, A. Jacobsen, J. Weimer, W. Arnold, C. von Haefen, D. Niederacher, R. Schmutzler, N. Arnold, S. Scherneck, *Oncogene* **2005**, *24* (5), 869.

[2] E. D. Wrenn, B. M. Moore, E. Greenwood, M. McBirney, K. J. Cheung, *J Mammary Gland Biol Neoplasia* **2020**, *25* (4), 337.

1. a) Values in the parenthesis are used in model variations in the supplementary figures. [↑](#footnote-ref-2)
2. b) Estimated from Ref [1] S. Seitz, R. Frege, A. Jacobsen, J. Weimer, W. Arnold, C. von Haefen, D. Niederacher, R. Schmutzler, N. Arnold, S. Scherneck, *Oncogene* **2005**, *24* (5), 869. [↑](#footnote-ref-3)
3. c) Estimated from Ref [2] E. D. Wrenn, B. M. Moore, E. Greenwood, M. McBirney, K. J. Cheung, *J Mammary Gland Biol Neoplasia* **2020**, *25* (4), 337. [↑](#footnote-ref-4)
4. d) Scaled down from spheroid parameters, based on the length scale from Figure 2h.

   ^e)^ Used to calculate the diffusion coefficient, *D.* [↑](#footnote-ref-5)
